# What do we know about nursing practice in relation to functional ability limitations, frailty and models of care among older people in home- and facility-based care: a scoping review

**DOI:** 10.1186/s12912-025-02948-7

**Published:** 2025-04-10

**Authors:** Ida Røed Flyum, Edith Roth Gjevjon, Anna Josse Eklund, Gunilla Borglin

**Affiliations:** 1 Department of Bachelor Education in Nursing, Lovisenberg Diaconal University College, Lovisenberggata 15B, Oslo, NO-0456 Norway; 2 Department of Nursing, Faculty of Health, Nature and Technical Science, Institute of Health Sciences, Karlstad University, Universitetsgatan 2, Karlstad, 651 88 Sweden; 3 UiT The Arctic University of Norway, Havnegata 5, Harstad, 9404 Norway

**Keywords:** Descriptive analysis, Frailty, Functional ability limitations, Functional decline, Long-term care, Nurse, Nursing practice, Older people, Registered nurse, Review

## Abstract

**Background:**

Nursing practice in long-term care, must support the delivery of safe and evidence-based care, especially for older people with functional ability limitations and frailty, with the competency, knowledge and structured working modes such practice requires. Understanding, detecting and preventing these conditions is important in a context where care is given to a significant number of older people with complex care needs. Our aim was to map published literature on how functional ability limitations and frailty among older people (65 and above) in home-and facility-based care (i.e. long-term care) were described by key stakeholders, and to identify models of care (MoCs) targeting these conditions.

**Methods:**

We followed Arksey and O’Malley’s methodological steps and the PRISMA-ScR reporting guidelines. The PubMed, CINAHL and PsycInfo databases were used to identify papers published between June 2002 and June 2022. The search was updated in May 2024. A descriptive analysis was conducted where the identified patterns were organised and categorised with the support of the Pattern, Advances, Gaps, Evidence for practice and research Recommendations framework (PAGER).

**Results:**

A total of 18,875 unique records were identified. Of these, 26 papers were included. The findings implied a discrepancy between the older people’s subjective- and the nurses’ more objective, ‘matter-of-fact’ perspective. The older people described both conditions in terms of identity loss and an emotional struggle to remain independent. They also highlighted the importance of positive connotations in relation to their efforts to adapt and accommodate the situation to the conditions. Nursing practice targeting the conditions were predominantly described as being reactive, based on their experiences and guided by ‘intuition’. The identified MoCs mainly targeted functional ability limitations while focusing on educating nurses.

**Conclusion:**

A point of saturation seems to have been reached regarding research focusing on older people’s descriptions of frailty in home-based care. The same cannot be said about older people’s or nurses’ descriptions concerning functional ability limitations or MoCs. Intervention studies focusing on nursing practice and the development of MoCs that target these conditions preferably in a home-based care context could substantially benefit the development of knowledge within nursing and nursing practice.

**Trial registration: Open Science Framework:**

10.17605/OSF.IO/FNHSA.

**Supplementary information:**

The online version contains supplementary material available at 10.1186/s12912-025-02948-7.

## Introduction

Among healthcare professionals, nurses, both registered and nonregistered, constitute the largest professional group (see Table 1 for operationalisations of key concepts). They are often the first point of contact, and they are also the group that spends the most time with patients. In addition, nurses are expected to provide a diverse range of healthcare services, where home- and facility-based care (i.e. long-term care) are two important arenas. One of the responsibilities and functions of registered nurses’ (RNs) clinical practice is to make clinical decisions based on a systematic process of assessments, diagnoses, plans, implementations and evaluations (i.e., the nursing process) related to the patient’s personal health needs [1, 2]. While nonregistered nurses can be conceived as the operational arm of RNs, as they carry out nursing functions under supervision and leadership from RNs [2]. Therefore, all nursing staff play a major part in delivering safe and evidence-based practice that involve detecting and establishing appropriate care actions. The latter is vital because home- and facility-based services, especially in the Nordic countries, are often described as necessitating more complex patient care, particularly for the increased number of older people presenting with chronic diseases [3].

**Table 1:** PICoS framework and operationalisation of core concepts

Criteria	Determinants (Inclusion criteria)	Operationalisation of core concepts
Population	Key stakeholders, here: Nurses, older people and significant others	Nurses or nursing staff, are operationalised as follows: Registered and nonregistered nurses, e.g., registered nurses, licensed practical nurses, nursing aides or healthcare assistants [31] Older people are operationalised as follows: People who are 65 years or older Significant others are operationalised as follows: Individuals with a close relationship to the older person, not necessarily defined by kinship or by being an unpaid carer
(Phenomenon of) Interest	Descriptions of functional ability limitations and/or frailty among older people Models of care targeting functional ability limitations and/or frailty in relation to older people	Functional ability limitations are operationalised as follows: A new loss of independence in self-care activities or as a deterioration in self-care skills [32, 33] Frailty is defined as: ‘a state of vulnerability to poor resolution of homoeostasis after a stressor event and is a consequence of cumulative decline in many physiological systems during a lifetime’ [34, p. 1] Models of care are operationalised as follows: A map of care (i.e., nursing- activities and/or interventions) aiming to safeguard that the older person with complex (care) needs receive the right care, from the right person and at the right time, hence outlining the best practice of care [19]
Context	Home- and facility-based care	Home- and facility-based care is operationalised as follows: Healthcare delivered over prolonged periods of time in the home or in a facility, i.e., home health nursing, residential aged care facilities, nursing homes or similar [35–37]. Please note that long-term care is at times used synonymously with these two concepts in the text
Study design	All study designs	

Statistics imply that the prospective number of older people in long-term care is estimated to increase from approximately 31 million (2019) to 38 million by 2050 [4]. Thus, the increased proportion of older people receiving home- or facility-based care has been well acknowledged [5]. In Norway alone, figures indicate that home-based care is the service that is increasing most rapidly [6]. The majority of older people needing these services are described as valuing their independence and, as such, preferring to remain in a familiar environment [7, 8]. Many are and will be living with multimorbidity which can be understood as the cooccurrences of two or more chronic conditions, including physical and/or mental health conditions, thus, exhibiting complex care needs [9]. Importantly, older people in need of home- or facility-based care are not a homogenous population. Instead, they are known to range from being relatively independent and in need of low-intensity care to being dependent and in need of high-intensity care [10].

In the group of older people receiving care in these contexts, it is not uncommon to present with functional ability limitations, and/or with some degree of frailty (Table 1). Additionally, we know that there is a considerable overlap between frailty and physical disability [11]. Even though more research is warranted to determine how for example physical conditions are associated with functional status (functional ability) and frailty and to determine factors that negatively influence frailty over time [12]. The latter is especially associated with several adverse health outcomes, such as falls, hospitalisation, increased healthcare-related costs, and death [13, 14]. In addition, many older people are still cohabiting with their partner and/or receiving support from them or from significant others [3, see 15]. Hence, it is fair to postulate that through them the health deterioration of older persons might be compensated for and delayed over a longer period of time, thereby masking their actual care needs, which can increase the risk of adverse health outcomes. Recognising frailty should be a vital part of nursing practice because frailty is both preventable and reversible [16]. Consequently, nurses play a vital part in ensuring that older people receive the correct interventions in relation to proactive prevention and treatment strategies. Being able to fulfil such responsibilities requires both qualified and competent professionals [17]; hence, a nursing practice characterised by systematic and evidence-based working modes. There is an undeniable need for structured working modes, such as effective models of care (MoCs), for the early detection and prevention of functional (ability) limitations and frailty among older people. The research into nursing practice implies that delivering care through distinctly articulated and defined MoCs can support nurses in working systematically towards a collectively decided set of goals [18]. However, it also aids nursing staff in their assessment and evaluation of care and supports nurses in sharing a joint foundation for care as well as sharing the same ‘picture’ of the given care. MoCs should here be understood as a map of nursing care (activities and/or interventions) aiming to safeguard that the older person with complex (care) needs receive the right care from the right person at the right time, hence outlining the best practice of care [19].

In summary home- or facility-based care services, for older people with complex care needs require strategies to detect signs and symptoms as well as prevent deterioration. This fits well within the remit and range of RN authorisation, responsibilities and function (professional scope of practice). However, the identified reviews imply that research within the area has focused mainly on screening tools and the effects of interventions [20–25]. Few of them have identified nurse-initiated or -led interventions. Furthermore, we argue that to be able to develop applicable frameworks organising and outlining nurses’ scope of practice (MoCs) related to these conditions, it is essential to gain in-depth knowledge about both conditions, hence advancing beyond our medical understanding. Therefore, the present study aimed to map published literature on how functional ability limitations and frailty among older people in home- or facility-based care were described by key stakeholders, and to identify models of care targeting these two conditions.

## Methods

Our scoping review followed the five steps of Arksey and O’Malley’s methodological framework [26], which is appropriate for broad and complex questions. In accordance with this framework, we have mapped our findings and identified gaps in published research. The PAGER (patterns, advances, gaps, evidence for practice and research recommendations) framework [27] was used for the visualisation (patterning) and reporting of the findings. The review is the first strand in a tier of four consecutive project strands [28], where the two upcoming strands will inform the development of an intervention targeting nursing practice related to functional ability limitations and frailty in the long-term care context, which will be tested in a fourth and final strand. The review adhered to the Preferred Reporting Items for Systematic Reviews and Meta-Analyses Extension for Scoping Reviews (PRISMA-ScR) checklist [29] (Additional file 1) and is registered (10.17605/OSF.IO/FNHSA) and preceded by a study protocol [30].

### Stage 1: identifying the research question

In agreement with the framework [26], we formulated our questions to the literature utilising PICoS—population, phenomenon of interest, context and study design to align them with our main search terms (Table 1). Based on experiences from the published protocol [30], the increased familiarity gained during the limited initial scoping search and on recommendations, we refined wording(s) and the core concepts. For example, long-term care was replaced by home- and facility-based care, functional decline was replaced by functional ability limitations, and our operationalisation of MoCs was simplified. The revised questions were as follows:

1. How is the condition of functional ability limitations among older people in home-or facility-based care described by key stakeholders?
2. How is the condition of frailty among older people in home- or facility-based care described by key stakeholders?
3. What models of care (nursing activities and/or interventions) can be identified as targeting functional ability limitations or frailty in relation to older people in home- or facility-based care?

In addition, we considered (I) by whom the questions were answered, (II) in which context(s), (III) in relation to whom or what, and (IV) which research design(s) was utilised.

### Stage 2: identifying relevant studies

Systematic searches were conducted in PubMed, CINAHL and PsycINFO. The search strategy was tested and evaluated with the support of an information specialist (Additional file 2). PubMed was used to develop our search strategy, which was then customised to each individual database. The first author (IRF) developed the first draft of the strategy and conducted an initial limited scoping search. The strategy was subsequently evaluated together with the last author (GB), yielding some minor adjustments. An iterative process preceded the final strategy. Search blocks were developed and included thesaurus terms, MeSH and keywords, including synonyms. These were combined via the Boolean operators AND/OR [38]. Limits were set to include English peer-reviewed primary research published during the last approximately 20 years (June 2002–June 2022). An updated search was conducted covering the date from the last search up to 27.05.2024.

### Stage 3: study selection

**Fig. 1:**
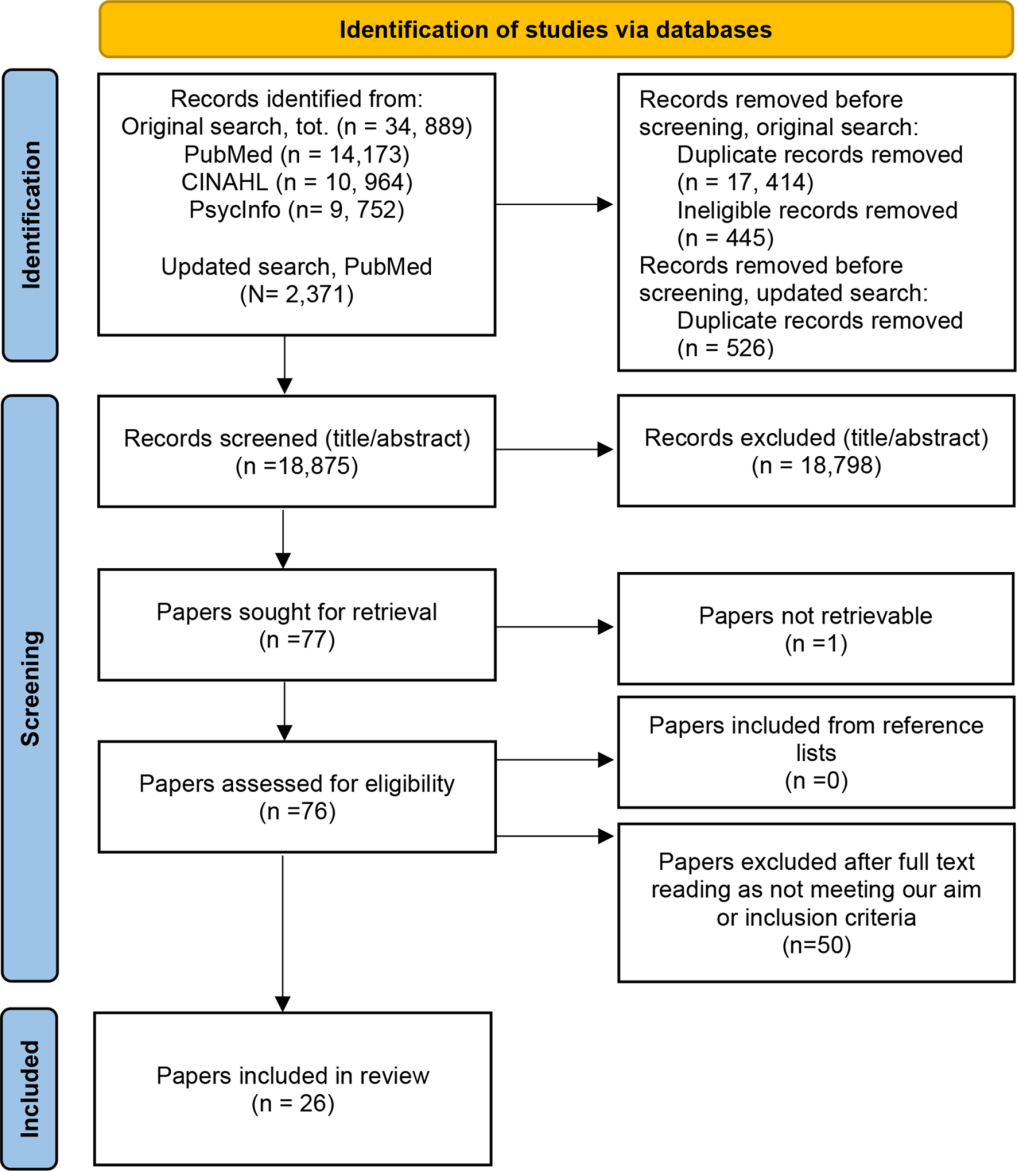
PRISMA flow diagram

The identified records were transferred to EndNote [39]; here, we removed duplicates before entering them into Rayyan [40] for title–abstract screening. All the authors (IRF, ERG, AJE and GB) conducted a joint title–abstract screening test guided by the PICoS determinates (Table 1). The records were screened by two independent reviewers to ensure agreement on selection. The first (IRF) and last author (GB) thereafter conducted a stepwise title–abstract screening, ‘sifting’ through eligible records [41] (Fig. 1 [42]). Disagreements were resolved through discussion and, if necessary, by consulting a third reviewer. Papers meeting the inclusion criteria were read in full text, and those with unclear relevance were assessed by two reviewers [43]. The reference lists of the included papers were screened, and no new papers were identified.

### Stage 4: charting the data

To facilitate systematic charting of the data, the first author (IRF) and last author (GB) developed a data extraction sheet (Tables 2, 3, 4); this was independently tested on six included papers [44]. Our testing resulted in minor changes related to the layout and level of charted detail. The first author (IRF) was mainly responsible for extracting data, whereas the last author (GB) randomly checked the accuracy and level of detail of the extracted data. The following information was extracted:

- Author(s), year of publication, country of origin
- Aim(s) of the study
- Context (home- and/or facility-based care)
- Study population (registered and nonregistered nurses; older people; significant others)
- Methodology (design, data collection and analysis)
- Models of care (nursing- activities and/or interventions targeting functional ability and/or frailty)
- Vital findings, i.e., answering our questions to the literature

**Table 2:** Data extraction Q1_Functional ability limitations

Author, year, country	Aim/research question(s)	Setting and participants	Design, data collection and analysis, categories/themes	Findings	**Sorting of findings**^**1**^ (Breaking down the text, inspecting and searching for patterns, assigning an identifier)	Categories
Imaginário, et al. [45] Portugal	To evaluate and establish a link between functional capacity and the different types of self-care profiles in a random sample of older people living in SCCs	Facility-based care 25 senior care centres, in the interior North of Portugal 313 older people (range = 65–104 years, mean = 83.44, SD = 7.12, ♀ = 201)	Transversal exploratory study, quantitative in nature, primarily relating to a nonexperimental plan Random sampling *Instruments:* The Portuguese version of the Barthel Index; the Lawton and Brody Scale, the Self-Care Dependence Evaluation Form (SCDEF), the Self-care of Home Dwelling Elderly (selfcare subscale profiles) and a questionnaire on demographic data. *Measures:* The degree of independence in performing the basic activities of daily living (BADL); Evaluate the functionality in the IADL; dependence in the activities of self-care was assessed; the instrument described four self-care profiles (responsible, independent, formally guided and abandoned) IBM SPSS (version 22) Descriptive statistics, parametric analyses, one-factor univariate analysis of variance (One-Way ANOVA, with a design for independent groups), The homogeneity test of variances of Levene, the Games–Howell test, manual calculation of the adjusted omega squared formula	This study showed that older people exhibited several levels of dependence on different self-care items. Bathing is the self-care activity most significantly impaired, while feeding represents the activity of self-care where patients show greater autonomy. Furthermore, staff were perceived to frequently take over tasks that the older persons could still manage themselves, such as managing prescribed medications, regardless of the individual’s level of autonomy or ability. The result indicates a need to implement recreational activities for older people, adapted to personal preferences, alongside the stimulation and motivation to participate. Related to the self-care profiles, the study found that the participants included in the abandoned self-care profile had significantly lower capacity to perform the BADL and IADL than the other self-care profiles, presenting a more negative prognosis in relation to the loss of functional capacities. The independent profile presented the highest capability, although this difference was not statistically significant compared to the responsible profile, implying that both the independent and responsible profiles are the most adaptive in terms of maintaining their functional capacities for a longer period. The results also highlight the lack of statistically significant differences in the functionality of the older people in the responsible and the formally guided profiles. The differences can be explained by the features of the self-care profiles	Capacity in (instrumental) activities of daily living is related to the self-care profile of the older person. The self-care activity in which older individuals experienced the most impairment was bathing, whereas they exhibited the most autonomy in feeding. [**Descriptive label:** Functional ability limitations and its “simple” descriptions] Regardless of the ability and autonomy of the older people the staff replaced them in several self-care tasks [**Descriptive label:** Functional ability limitations and the “well-intended” nursing practice] Recreational activities customised to the older persons preferences and including appropriate motivation for these activities is indicated as important [**Descriptive label:** Functional ability limitations and its relation to everyday life]	Notions—Justifications Implications—Nursing practice Implications—Everyday life
Lehto-Niskala et al. [46] Finland	To explore the role of family members in long-term care, and in particular, their ways to support the functional ability of their older relatives	Facility-based care Eight care facilities (two nursing home wards, two long-term hospital wards, and four assisted living facilities with 24-hr care) in two municipalities in southern Finland. Two of the facilities were public and six were run privately 16 family members of LTC residents (range = 48–77 years, ♀ = 13) The participants had their mother (n = 9), father (n = 1) or spouse (n = 6) living in the facility [Mean age not specified]	Qualitative design Semi-structured interviews Thematic analysis **Theme:** Engaging in daily activities with the *sub-themes*; Organising care; Providing physical support; Taking part in care decisions **Theme:** Monitoring care with the *sub-themes*; Prior experiences influencing observations of care; Filling observed gaps in care **Theme:** Bringing forth personal needs and wishes with the *sub-themes*; Pointing out their relative’s likings; Providing meaningful social contacts	Family members supported the functional ability of their older parent or spouse by organising and monitoring care and by bringing forth their relative’s personal needs and wishes. The majority described their involvement and participation in various activities as important and even a matter of necessity. A sense of dissatisfaction and disappointment about care quality led to family members assuming greater responsibility for care provision themselves and filling the gaps in care. Some related this to lack of resources. They often saw their role alongside staff members as ambiguous, and their understanding of the scope of support for functioning extended beyond physical everyday tasks. In their talk, family members broadened the concept of functional ability from daily chores and independence to meaningful social relations, emotional needs and acknowledgement of person’s individual background and preferences. The findings show that maintaining personhood is as an important part of maintaining functional ability. Some family members described believing improvement of functional abilities is possible	Functional ability, and its maintenance, is described by family members as including more than solely ADL and independence, not least achieving meaningful social relations, emotional needs and maintaining personhood through acknowledgement of the individual background and preferences [**Descriptive label:** Functional ability limitations and its relation to everyday life] Improving functional abilities were believed as possible by some family members [**Descriptive label**: Functional ability limitations and it´s reversibility] The family members saw their role in supporting functional ability as ambiguous next to the care staff, but still as important and sometimes even a necessity due to lack of resources [**Descriptive label:** Functional ability limitations and challenges in nursing practice]	Implications—Everyday life Notions—(Pre)conceptions Implications—Nursing practice
Letho et al. [47] Finland	To explore the meanings given to functional ability in the interview talk of long-term care nurses and older people living in long-term care	Facility-based care Eight different LTC facilities (two nursing home wards, two long-term hospital wards, and four assisted living facilities with 24-h care) in two cities in southern Finland. Two of the facilities were public and six were run privately 24 nurses: 5 RNs 19 practical nurses (♀ = 23) 16 older people (♀ = 11) [age not specified]	Qualitative design Semi-structured interviews Discourse analysis **Result headings:** The nurse as a competent professional and active caregiver; LTC residents as active individuals and recipients of help	There are differences in how nurses and older people understand functional abilities. Nurses’ understanding differed related to how they positioned themselves, as an active caregiver or as a competent professional. Functional ability was about the basic functions of everyday life, related to being independent or dependent, and physical and psychological well-being. Alternatively, functional ability commonly took shape either as a formal, standardised indicator or an abstract combination of physical, social, and psychological domains. Being active was promoted. Some hesitation among the nurses related to their understanding of functional ability was also noted and their talk was often formal and theoretical and seemed heavily influenced by official care policies and textbooks. Residents understood functional ability as a more versatile concept. Their understanding was typically based on their previous experience, and differed depending on which position it was understood from; an active individual taking care of him or herself, a recipient of help, or a burden to nurses. To an active individual with reduced functional abilities, it represented the effort to cope with health problems. Residents also related functional ability to the need for help, and activity/independence were positive aims. In contrast to the nurses, residents also mentioned activities beyond daily chores, including writing, drawing, and watching television. Being dependent or independent was not a straightforward dichotomy; rather, it was about different ways of coping with functional problems. Residents compared their functional ability with previous capacity and what it might be in the future, this time dimension was missing from the nurses’ understanding. The residents emphasised the scarcity of nurses, thus they tried to ease nurses’ work by being as independent as possible. Importantly, the residents resisted the position of solely passive care receiver and also talked about functional ability as a factor that one could influence	The older people and the nurses understood functional ability in different ways. Nurses perceived it primarily in two distinct ways; first related to everyday tasks, being dependent or independent second as an abstract term including physical, social, and psychological domains or as a standardised indicator. The older people understood functional ability as different ways of coping with functional difficulties, rather than a dichotomy of independency or dependency. Functional ability was hence understood by the older people as a versatile concept, needing help, coping with difficulties, to feeling like a burden [**Descriptive label:** Functional ability limitations, a challenging process] (In)dependence and activity was related to functional ability among both the older people and the nurses [**Descriptive label:** Functional ability limitations and its “simple” descriptions] Pleasurable activities were highlighted as important related to functional abilities by the older people, in addition to daily chores [**Descriptive label:** Functional ability limitations and its relation to everyday life] The older people resisted the label of being solely care receivers and believed they could influence their functional ability [**Descriptive label:** Functional limitations and it´s discord (in R/T older people)] The older people tried to be as independent as possible to ease the burden of the already pressed nurses [**Descriptive label:** Functional ability limitations and challenges in nursing practice]	Notions—Justifications Notions—Justifications Implications—Everyday life Notions—(Pre)conceptions Implications—Individual level
Palacios-Ceña et al. [48] Spain	To describe how dependence was experienced by Spanish nursing home residents with functional limitations	Facility-based care Five private, for-profit nursing homes located in the southern part of the Greater Madrid area, Spain 30 residents, (range = 62–100 years (2 residents < 65), mean = 83 years, SD = 9, ♀ = 15) 10 of the residents were interviewed twice (reason given: interruptions by visitors, exhaustion, and one case of a medical situation)	Qualitative phenomenological study Unstructured and semi-structured interviews 10 personal letters and two diary fragments Field notes, the Giorgi proposal **Theme:** Remaining “capable” with the *sub-theme*; Building the difference **Theme:** Sharing life with the *sub-themes;* Living with “non-capable” residents; Sharing the environment	The findings from this study showed that residents themselves had made types of dependence classifications, based on their environment and beliefs. This gradation of capability is different from typical professional criteria, it had nothing to do with needing assistive devices or support in daily activities. The findings suggest that it is difficult to avoid the “non-capable” label and that this was a label the resident could have “forever”. That is why residents try hard to remain “capable”, not only for health reasons, but to avoid exclusion. It also appears that “capable” residents interact better, are more able to express their opinion and to relate to others. They seem to receive certain privileges from the staff. Thus, it appears a sort of discrimination against dependence. In fact, some residents, despite having functional limitations and difficulties when carrying out certain activities, do not perceive themselves as non-capable. In the study, being unable to walk independently was perceived by residents as the first step to dependence. Hence, many residents were not willing to use assistive devices, not even on a temporary basis. Furthermore, there was a tendency for independent residents to avoid sharing the same environment (e.g. a room, a table when having a meal) and spending time with dependent residents, due to not wishing to be identified as having a disability. It appears that residents considered “non-capable” might request more professional care and higher support to adapt to daily life	Being “non-capable” did not relate to needing support in daily activities nor using assistive devices. Not being able to walk was seen as potentially the first sign of dependence, and hence, showing any signs of such was resisted [**Descriptive label:** Functional ability limitations and its “simple” descriptions] Particularly negative attitudes towards being dependent (“non-capable”) and the potential for being excluded made the older people fear and work hard to not be labelled as such. Causing the older people to resist the potentially permanent label, even when having functional limitations. Discrimination against those labelled as “non-capable” seemed to exist, also in how the nursing staff acted [**Descriptive label:** Functional limitations and it´s discord (in R/T older people)]	Notions—Justifications Notions—(Pre)conceptions
Sacco-Peterson and Borell [49] Sweden	To generate a deeper understanding about how physical and socio-cultural environments and individuals’ cultural beliefs influence residents’ participation in their personal-care activities	Facility-based care A women’s ward of a nursing home in a Southern European country Observations: 49 residents living on the ward (range = 52–97 years (3 residents < 65), mean = 81 years, ♀ = 49) Permanently assigned care staff: Head nurse and deputy head nurse 4 registered nurses 7 nursing aides Informal conversations: e.g., nursing and cleaning staff, residents, and families [age/sex not specified] In-depth interviews: 9 older people (range = 68–85 years, ♀ = 9)	Ethnographic design using both qualitative and quantitative methods Participant observation and narrative interviews through informal conversations In-depth repeated narrative interviews A detailed mapping of the entire ward Constant comparative method Manifest content analysis using descriptive statistics **Categories:** The defeating geography of the ward; Diapers as a solution; Diapers as a dilemma; Physical environmental constraints to self-care; Socio-cultural constraints to self-care; Staffs’ responsibilities and residents’ expectations; Good intentions gone wrong	The findings demonstrate how the physical and socio-cultural environment in the nursing home required older residents to overcome greater physical and cognitive challenges to maintain their participation, autonomy, and dignity in toileting, bathing, and dressing than would have been expected had the resident been living at home. Nonetheless, despite environmental challenges, residents illustrated the value they placed upon their participation in self-care as they experienced tremendous daily struggles in order to do so. The results show that the older residents were not as institutionalised as would be believed. Paradoxically, because of their own socio-cultural beliefs, residents also constrained their independence, ease and participation in self-care. The study also highlights the dilemma facing the nurses knowing that they facilitated incontinence by diapering persons who were continent, but they were working without formalised care plans in increasingly chronic short-staffed conditions which caused the nursing staff to work in ways contrary to what they verbalised to be best practices. This, in turn, defeated the most vulnerable residents who struggled to maintain their dignity in self-care. Furthermore, the findings revealed that even staff who work intimately with residents can be unaware of daily unnecessary struggles in self-care that residents in nursing homes may be experiencing. The study also discovered that the outcomes of interventions by nurses and institutions, intended to facilitate self-care participation, were not always reflected upon nor assessed, but rather assumed to be effective. As a result, outcomes assumed to be achieved were not being realised**—**resulting in undiminished residents’ risk for falls and their continued struggles in self-care	Maintaining participation, autonomy, and dignity in activities of daily life is made increasingly difficult for older people due to the nursing home environment [**Descriptive label:** Functional ability limitations and challenges in nursing practice] Tremendous struggles in participating in activities of daily life was experienced by the older people, still they show that this is valuable, while at the same time they constrained their own independence [**Descriptive label:** Functional ability limitations, a challenging process] The older people could experience difficulties and need more support than the nursing staff were aware of, despite the close nature of their work. Working in accordance with the known best-practice was sacrificed due to contextual difficulties- potentially harming the older person self-care and dignity [**Descriptive label:** Functional ability limitations and challenges in nursing practice] Interventions with the intention of easing the older people’s participation in activities of daily life, but without evaluation or reflection could lead to expected outcomes not being achieved [**Descriptive label:** Functional ability limitations and the “well-intended” nursing practice]	Implications—Everyday life Implications—Individual level Implications—Nursing practice Implications—Nursing practice

^1^Adapted from Ritchey 1996

**Table 3:** Data extraction Q2_Frailty

Author, year, country	Aim/research question(s)	Setting and participants	Design, data collection and analysis, categories/themes	Findings	Sorting of findings^1^ (Breaking down the text, inspecting and searching for patterns, assigning an identifier)	Categories
Bjerkmo et al. [50] Norway	To explore how single-living frail older adults experience living with frailty in everyday life in rural Arctic areas	Home-based care Home care services in two rural municipalities in the northernmost county of Norway 8 older people, identified as frail (age range = 82–93 years,♀ = 6)	A qualitative longitudinal design A series of interviews Inductive thematic analysis **Theme:** Frailty as a phenomenon **Theme:** Frailty as part of old age with the *sub-themes*; Physical and mental decline; Letting go; Accepting the need for help; Being alone **Theme:** Frailty in a rural Arctic context with the *sub-themes*; Climate; Long geographical distances; Societal changes	The participants’ experiences of frailty varied over time. Becoming increasingly frail was partly a result of changes in health conditions. The participants shared experiences of “being almost in the reach of death” and then coming to life again. Many participants tried to adapt to the changing circumstances, while others found it more challenging. However, the findings are in line with that frailty is not a state of inevitable, “one-way”, progressive decline. Rather, they experienced “frailty” as dynamic state and as something they had to cope with, balancing losses and capacity in everyday life. Some described being frail for a shorter or a longer period of time, for example in connection to a fracture, where they could experience being frailer afterwards. Some associated frailty with having to accept more help. Their stories concerned tasks or skills that they had mastered but also those that they eventually had to let go of. Physical changes caused limitations in their everyday lives. Several participants made a distinction between physical frailty and mental frailty. Some linked frailty primarily to bodily changes and others to mental changes. Participants’ stories also concerned being alone. Not having someone to talk to was experienced as a loss, while others had come to terms with what life had become. The findings demonstrated that frailty cannot be understood solely as an individual trait or condition. Rather, shifts in the balance point (of frailty) result from an interplay between age, health related changes, contextual challenges, as well as the physical and social environments. The participants, in varies degrees, considered the experienced changes (frailty) to be a result or a part of increased age. Several tried to adapt to the changes, while others found it more challenging to accept the limitations that growing older had created. The findings showed that the rural Arctic environment affected the participants’ experiences of frailty (snow, distances, etc). Changes in society also contributed to their experiences of frailty, such as the closing of the local bank and transition to digital solutions made the participants dependent on others to perform tasks that they had previously mastered. The participants also acknowledged that their own frailty limited their social interaction. Several participants expected that they would have to move from their own homes in the future as a consequence of increased care needs and the need for social contact. However, most participants wanted to continue living in their own homes and communities for as long as possible, despite challenges	Frailty was by the older people experienced as a dynamic state where they had to balance losses and capacity in their everyday life. There were differences in the ability to adapt. Physical challenges, together with contextual, societal changes and the physical and social environment produced limitations in the older people’s everyday life. However, despite challenges, most older people wanted to live at home, simultaneously expecting to have to move. The older people experienced being alone as challenging in different degrees, some experienced it as a loss, while others had reconciled with the changes. Their own frailty was recognised to limit their social life [**Descriptive label:** Frailty, a constant process to defy, defeat conquer] Several older people made a division between types of frailty, connecting frailty to physical changes or mental changes. The older people’s experiences described frailty not as exclusively an individual trait or condition, but rather as an interplay between age, health related changes, contextual challenges, as well as the physical and social environments. Frailty was experienced as connected to increasing age in different degrees by the older people. Some adapted to these changes, while other found it difficult to accept [**Descriptive label:** Frailty and its relation to functional ability]	Implications—Everyday life Notions—Justifications
Britton [51] England	To explore the experiences of community nurses in assessing frailty and planning interventions around frailty	Home-based care Community service provider in West of England. 6 community nurses [age/sex not specified]	Qualitative design Semi-structured interviews Thematic analysis **Themes**: Aspects of frailty; Frailty as an emerging concept; Lack of confidence with assessment tool and lack of certainty around frailty; Nursing knowledge and intuitive knowing; Barriers to assessing frailty within community nursing; Suggestions going forward	Participants discussed several key aspects associated with frailty such as appetite, medication and polypharmacy, past medical history and long-term conditions, social and family support, cognition, environment, and equipment use. One key characteristic raised in all interviews was mobility. Participants discussed that they routinely look at patients’ mobility and walking pace, using opportunities, such as when a patient answers the door, to assess walking. Generally, participants linked frailty with older age. Some participants discussed an intuitive feeling, intuitive knowing, that patients are frail and while they discussed some key aspects of frailty, they were not able to comprehensively verbalise the rationale behind this ‘feeling´. Some uncertainty around the concept of frailty and its definition was noted and some called for more in-depth training. Participants had a growing awareness of frailty in practice, but challenges such as time pressures, described their job as ‘busy, short staffed’ and ‘high pressured’. Participants linked time constrains with difficulty in assessing frailty. Participants discussed that more staff and more time would make frailty assessment more achievable. The Rockwood frailty scale was used within practice, but it was perceived to lack validity within the community setting. It was also discussed that nurses sometimes perceived some gaps within services, preventing them from referring on. Comments regarding patients ‘slipping through the net’	Described key-aspects used for observation and assessment by the nurses [Descriptive label: Frailty and its “simple” description] Professionals experienced an uncertainty around frailty and its definition [Descriptive label: Frailty and its ambiguity] Lack of knowledge and calls for in-depth training [Descriptive label: Frailty and the lack of nursing knowledge] Frailty equals older age [Descriptive label: Frailty and its negative connotations] Nursing practice focusing on the detection/identification of frailty was guided by an intuitive feeling without nurses having the ability to verbalise the rationale behind this feeling [Descriptive label: Frailty and its intuitive nursing practice] The nurses’ ability to assess frailty was negatively influenced by organisational issues such as time pressure and lack of staffing and gap in services, but also by the instrument meant to be implemented for assessment which were experienced as not being relevant for their setting [Descriptive label: Frailty and organisational challenges]	Notions—Justifications Notions—Uncertainties Notions—Deficiencies Notions—(Pre)conceptions Implications—Nursing Practice Implications—Nursing Practice
Lloyd et al. [52] United Kingdom	To understand the changing experiences of frail older people through the stories that they told	Home-based care Community-dwelling older people recruited from a medical day hospital 13 older people (age range 76–92 years, mean 86 years, ♀ = 8) [5 participants died during the study, ♀ = 4] 13 informal caregivers 8 case-linked professionals: 2 care workers 5 general practitioners 1 occupational therapy assistant [age/sex not specified]	Qualitative longitudinal approach Interviews (individually or jointly; older person and informal carer) Analysed using The Voice Centred Relational Method **Three distinct forms/patterns:** Narratives of stability and coping; Narratives of struggle and unbalancing; Narratives of becoming overwhelmed **Result heading** (not specified as a theme/category): Bringing the narratives together	The frail older people told stories of their experiences that revealed three distinct patterns. These were either stable, unbalancing or overwhelmed and related to how the person managed to adapt to increasing challenges and losses, and to reintegrate their sense of self into a cohesive narrative. Frailty was described as both biographically anticipated yet potentially biographically disruptive as older people may struggle to make sense of their circumstances without a clear single causative factor. The experience of living with frailty involved increasing losses which disrupted, and progressively restricted, the everyday lives of older people. This involved disruption of the taken-for-granted aspects of the physical body, of daily life and relationships with the self and others, where the person experienced a shift from their normal expected life trajectory that undermines self-identity. Varying degrees of fear and anxiety over what the future may hold was described	Described three distinct patterns of frailty: stability, unbalancing or overwhelming [Descriptive label: Frailty and its “simple” description] Frailty was expected but the experience of living with frailty was that frailty was expected but included varying degrees of adaption to growing difficulties and losses which disrupted, and progressively restricted, the everyday lives and relationships of the older people as well as a struggle to reintegrating their sense of self in this process. Living with frailty was described as involving varying degrees of fear and anxiety related to what may be in store for the future [Descriptive label: Frailty, a constant process to defy, defeat conquer] The older people also experienced a shift from their normal expected life trajectory that undermined their self-identity [Descriptive label: Frailty and its discord]	Notions—Justifications Implications—Everyday life Implications—Individual level
Nicholson et al. [53] United Kingdom	To understand the experience of home-dwelling older people living with frailty over time	Home-based care Community-dwelling older people recruited through an older persons’ intermediate care team 17 older people (age range 86–102 years, ♀ = 12)	Combined qualitative psychological method In-depth interviews using the Biographic Narrative Interpretative Method (BNIM), Free Association Narrative Interview Method (FAINM) and psychodynamic observations Analysed using a modified BNIM analysis and cross-case analysis Main themes: The dynamics of physical and psychosocial frailty; Sustaining connections within the home; Connecting with death dying	In the findings of this study frailty is presented as a persistent liminal state. The dynamics of physical and psychosocial frailty details the persistent state of uncertainty and loss experienced as a result of progressive physical and psychosocial changes. To retain anchorage in this state of imbalance, frail elders work actively to develop and sustain connections to their physical environment, routines, and social networks. The study also reveals the problematic nature of finding shared meanings between older people and health and social care professionals within the continual and shifting state of frailty. Important note, nobody in the study used the term ‘frail’ to describe themselves or their situation	Frailty was experienced as a persistent state signified by uncertainty and losses due to physical and psychosocial changes, to remain anchored older people worked to develop and sustain connected to routines, their environment and social networks [**Descriptive label:** Frailty, a constant process to defy, defeat conquer] Frailty was not used by the older people as a concept to describe themselves or their situation [**Descriptive label:** Frailty and its discord]	Implications—Everyday life Implications—Individual level
Nicholson et al. [54] United Kingdom	To understand the experience of home-dwelling older people with changing states of frailty	Home-based care Community-dwelling older people recruited through an older persons’ intermediate care team 15 older people (age range 86–102 years, ♀ = 10)	Combined qualitative psychological method In-depth interviews using the Biographic Narrative Interpretative Method (BNIM), Free Association Narrative Interview Method (FAINM) and psychodynamic observations Analysed using a modified BNIM analysis and cross-case analysis **Main themes**: Losses and disconnects within frailty; Sustaining connections; Creating connections	Frailty was understood in terms of potential capacity—a state of imbalance in which people experience accumulated losses whilst working to sustain and perhaps create new connections, as well as creating everyday routines. This process was overlapping and multifaceted. The participants demonstrated capacity to overcome or find others to overcome their physical, emotional, or social vulnerabilities. However, participants did all speak of loss over time, loss of physical capacity, social status, friends, and family. For most the opened discussions around dependency, finitude and their experiences of barriers related to societal and welfare systems. The narratives suggest that the balance between (loss of) autonomy and dependence and changing roles is complex. Nobody used the term ‘frail’ to describe themselves or their situation. The older peoples extraordinary work of relating their ordinary world in a different way does not equate to the predominant stereotypical image of frail older people which focus on vulnerability	Frailty was understood (experienced) as a state of imbalance where losses accumulated whilst the older person was working to sustain and creating everyday routines whilst still demonstrating capacity to overcome losses [**Descriptive label:** Frailty, a constant process to defy, defeat conquer] Frailty was not used by the older people as a concept to describe themselves or their situation. Instead, older people’s experiences and their capacity to handle the accumulated losses did not equate to the stereotypical image of frail older people which focus on vulnerability [**Descriptive label:** Frailty and its discord]	Implications—Everyday life Implications—Individual level
Obbia et al. [55] Italy	To explore the views and experiences of primary care professionals working with older people on of the concept of frailty Research questions: • What are the views held by primary care professionals regarding frailty in older people? • What are their experiences regarding the early detection of frailty among their older clients? • What are their experiences of introducing preventive interventions in practice with older people showing signs of frailty?	Home-based care Four local health agencies in one middle-sized town, one rural area, one affluent urban area, and one deprived urban area, in Piedmont, Italy 33 practitioners: 11 district nurses 8 home care workers 6 social workers 4 physiotherapists 4 GPs (mean age = 48, ♀ = 29)	Qualitative descriptive phenomenological design Focus group interviews; N = 4 Phenomenological analysis **Theme:** The psychosocial nature of frailty with the *sub-themes*; Loneliness; Financial issues; Family and community networks; Psychological distress; Hidden cognitive problems; Loss of independence **Theme:** Late detection **Theme:** The enablers/barriers to preventive interventions with the *sub-themes*; Support and related outcomes; Lack of awareness and fear of being labelled; The wall of bureaucracy; Access to the care network; Integration of care	Frailty was considered a constituent part of the ageing process, and the term frail was confused with the presence of known disability and multimorbidity and no clear distinction was made between the term’s frail, multimorbidity and/or disability. Implying the presence of a skills gap related to the detection of the early signs of frailty. The professionals experienced difficulties in identifying those who were frail or who may become frail. Early detection and effective preventive interventions were considered complex but feasible by participants. However, this may require a systematic restructuring of primary care organisations. Important dimensions of frailty were loneliness, financial issues, family/network, comorbidity, disability, hidden cognitive problems and phycological distress while family, neighbours, or informal caregivers were seen as “protective factors”. Participants believed the older people did not want to be labelled as frail or as someone in need	Professionals could not distinguish between frail, disability or multimorbidity [Descriptive label: Frailty and its ambiguity] Lack of knowledge and training related to early detection of frailty and the prevention of frailty [Descriptive label: Frailty and the lack of nursing knowledge] Frailty was preventable but the present health- and social care system and a lack of focus hindered such work [Descriptive label: Frailty and its reversibility] Frailty equals negative connotations of being old and in need of help [Descriptive label: Frailty and its negative connotations] Staff described that older people did not want to be labelled as frail or in need of help [Descriptive label: Frailty and its discord] Described protective factors as well as important factors of frailty [Descriptive label: Frailty and its “simple” description]	Notions—Uncertainties Notions—Deficiencies Implications—Nursing Practice Notions—(Pre)conceptions Implications—Individual level Notions—Justifications
Papadopoulou et al. [56] Scotland	To understand the perceptions of community nurses about frailty; training and use of educational materials on frailty; learning needs in relation to identification, assessment, and management of people with frailty; and their leadership role in interdisciplinary practice within community teams	Home-based care One health board area in Scotland Community nurses; N = 17 Team leaders, with formal specialist practitioner qualification, registered nurses, and clinical support workers [age/sex not specified]	Exploratory qualitative design Focus group interviews; N = 3 Individual interview; N = 1 (team leader) Thematic content analysis **Theme**: Concept of frailty with the *sub-themes;* Meaning of frailty; Process of caring for the frail **Theme:** Knowledge about frailty with the *sub-themes;* Educational needs; Building capacity in the context of adversity	The term frailty was associated with negative attributes, such as being vulnerable and old, experiencing various losses and having multiple health conditions**—**complex comorbidity. Loneliness and social isolation were also recognised as complicating frailty. The nurses differentiated between the inevitability of the ageing process and the potential for frailty to be reversible or preventable. They also acknowledged the potential for early interventions to prevent functional decline. Current practice was described as largely reactive, guided by experience and intuition, with little systematic frailty-specific screening and assessment rather than a systematic use of evidence-informed frailty specific screening and assessment. Thus, the nurses expressed a need for frailty-specific education, particularly around assessment	Frailty equals negative connotations of being old, and vulnerable [Descriptive label: Frailty and its negative connotations] Frailty was perceived as preventable and reversable with early interventions, but current practice was not organised to support this [Descriptive label: Frailty and its reversibility] Nursing practice focusing on the detection/identification of frailty was reactive and guided by intuition and experience [Descriptive label: Frailty and its intuitive nursing practice] Lack of knowledge about frailty and its assessment [Descriptive label: Frailty and the lack of nursing knowledge]	Notions—(Pre)conceptions Implications—Nursing Practice Implications—Nursing Practice Notions—Deficiencies
Skilbeck et al. [57] England	To explore how older people with complex health problems experience frailty in their daily lives	Home-based care A community matron team in a city in the North of England 10 older people (age range 77–91 years, median 84 years, ♀ = 7)	Ethnographic study Participant observation (up to six encounters per participant) Semi-structured interviews Constant comparative analysis **Theme:** Fluctuating ill-health and the disruption of daily living with the *sub-themes*; Ongoing disruption; Stability and disruption **Theme:** Changes to the management of daily living with the *sub-theme*; Keeping going **Themes:** Frailty as fear, anxiety, and uncertainty; Making sense of changes to health and daily living	The frail older people experienced transitions in health and illness as a continual process of change, with sudden events, general decline, and periods of relative stability. Older people work hard to modify and maintain daily routines to achieve balance. Sustaining daily routines and renegotiating activities and priorities contributed to the maintenance of an older person’s identity as an independent person. This occurred even when transitions were enduring and multifaceted in nature, often pushing an older person’s adaptation to the limit and striving to construct and conserve daily routines, the older people in this study did not live up to the stereotyping associated with frailty. Episodic moments of frailty occur, where daily living becomes precarious, and their resilience is threatened. This could culminate in moments of fear, anxiety, uncertainty, and feeling frail. Bodily decline often tips this balance. The older people seemed to anticipate a situation where independence may decrease and the ability to exercise personal agency is reduced, led older people to contemplate their ability to continue living at home, their own ageing and mortality. Older people were aware of what they could not do for themselves and how this fluctuated. However, they focused on continuing to engage with daily living and in doing so they defined themselves in relation to what they could achieve	Frailty was experienced as an ongoing process of transitions of health and illness characterised by general decline and relative stability which forced older people to work hard to maintain their daily life and achieve a balance to continue their identity as independent while they continued to focus on what they could do. When this process failed and the adaption was pushed to the brink the older people experienced fear, anxiety, uncertainty and feeling frail [**Descriptive label:** Frailty, a constant process to defy, defeat conquer]	Implications—Everyday life
Strømme et al. [58] Norway	To develop knowledge about homecare professionals’ observational competence in early recognition of deterioration in frail older patients Research question: • How can homecare professionals’ practices and experiences with early recognition of deterioration in frail older patients be described?	Home-based care Two home-care districts in two municipalities in western Norway; city, urban and rural areas. Focus group interviews; N = 30 Registered nurses, skilled health workers and assistants [age/sex not specified]	Explorative, qualitative mixed methods design Participant observation; 62 h Focus group interviews; N = 6 Qualitative content analysis **Theme:** Patient-situated assessment of changed clinical condition with the *sub-themes*; Knowledge of the patient; Changed physical and mental function; Basic understanding of vital signs **Theme:** Organisational environment with the *sub-themes*; Focus on planned practical tasks; Collaboration, and collegial support	Awareness of, and ability to recognise signs of deterioration varied and was at times quite low. No clear difference was found between the RNs, skilled health workers and assistants in noticing early signs of deterioration. Monitoring and measuring patient’s vital clinical signs were not a priority among staff. Thus, vital signs were in general measured infrequently. When a patient’s situation was vague or critical, vital signs were measured more frequently. In situations where nonspecific signs and symptoms were the only indicators of a patient’s decline the HCPs emphasised the importance of knowing the patient. Many found it difficult to visit unfamiliar patients and assess their clinical conditions. In a few situations, changes in physical and mental functioning led to the HCPs communicating with the patient and monitoring certain vital signs. However, in most instances, HCPs described relying on intuition and feeling a sense of concern to pinpoint signs of decline. The organisational environment influenced their practices, their routines were described in detailed workplans which affected their assessments of the patients’ decline. The HCPs expected actions and tasks during home visits to be part of the detailed work plans	Lack of knowledge and skills in early recognition of deterioration among both RNs and other Health Care staff. Both groups did not show visible differences related to how they noticed early signs of deterioration [**Descriptive label:** Frailty and the lack of nursing knowledge] Nursing practice of early recognition of deterioration were guided by the HCPs intuition and sense of concern and monitoring or measuring vital signs were not prioritised [**Descriptive label:** Frailty and its intuitive nursing practice] HCPs practice, actions/tasks were described as controlled by set workplans not supporting early recognition of deterioration and the non-specific signs and symptoms aggravated the HCPs ability for early recognitions. Early recognition of deterioration demanded knowledge about the patient [**Descriptive label:** Frailty and organisational challenges]	Notions—Deficiencies Implications—Nursing Practice Implications—Nursing Practice
Søvde et al. [59] Norway	To explore the lived experiences of frail home-dwelling older people Research question: • How do home-dwelling older people experience frailty?	Home-based care Two geriatric outpatient clinics 10 home-dwelling older adults (age range 72–90 years old, ♀ = 7)	Hermeneutical phenomenological study In-depth interviews Hermeneutic phenomenological **Theme:** Frailty as being in the borderland of the body with the *sub-themes*; The body shuts down; Living on the edge; Not giving up	The experience of frailty is described as an ambiguous experience of balancing frailty, strength, and an altering body. Given the findings of this study, frailty might be experienced as a downward spiral of losses of physical functioning, social engagement and a pervasive risk of injury when performing daily activities. Participants even started questioning their capability to walk on stairs without falling or get to the door when the doorbell rang without tripping over their feet. The findings show that participants experienced that they could not escape or recover from frailty, and some had to give up meaningful activities, which was a big loss. Still, participants adjusted previous activities to keep up meaningful activities, and still being independent, this strengthened their feeling of being themselves. They used past experiences of overcoming life challenges to endure and to hold onto what was most important for them. Participants described fear of being left with a meaningless everyday life with nothing to fill the days	Frailty was described and experienced as a downward spiral of losses from which they could not escape or recover from, still using past life experiences and adjustment of their daily activities supported them to experience independency and being themselves. The spiral of losses meant that frailty could be accompanied by a fear of being left with a meaningless everyday life [**Descriptive label:** Frailty, a constant process to defy, defeat conquer]	Implications—Everyday life
Voie et al. [60] Norway	To explore how home care professionals conceptualise frailty in the context of home care	Home-based care One large municipality in Northern Norway 14 Registered nurses and certified nursing assistants home care or day care centres (♀ = 11) [age not specified]	Qualitative research design Focus group interviews; N = 4 Thematic analysis **Themes:** “Frail”–a term which is too imprecise to be useful; Frailty as a consequence of ageing; Frailty as lack of engagement and possibilities for engagement; Frailty as a contextual phenomenon; Frailty as potentially affected by care	The home care professionals conceptualised frailty as an individual trait but also as resulting from the interplay between individual and environmental factors. Moreover, the home care professionals conceptualised frailty diversely; representing a continuum between frailty as related to prevention and management (‘cure’), and frailty as related to ageing as natural decline (‘care’). The participants thus conceptualised frailty in accordance with the service users’ diverse health and care needs, and how they as home care professionals provide services that range from supporting people with minor tasks, such as medication delivery and domestic care, to caring for people with extensive health and care needs. Furthermore, the terms frail and frailty were considered ‘too imprecise to be useful’ while also being terms to which the home care professionals ascribed several contrasting meanings. Rather than using the term ‘frail’, the home care professionals in this study preferred to use terms they considered more ‘professional’ and specific when addressing service users’ care needs. The participants in this study acknowledged that frailty is ‘potentially affected by care’ and considered physical activity, nutritional support and social support as means to prevent or reduce frailty. The participants conceptualised frailty as both a natural age-related decline, which could be expected in very old age, and as a state that can be prevented or reduced. The results indicate that while home care professionals struggle to conceptualise frailty, they manoeuvre the continuum between cure and care in their everyday practices and in encounters with older persons with complex care needs. While the home care professionals talked about interventions to prevent and reduce frailty, statements also demonstrated that they also recognised the limits of curative models of care and that frailty is not always possible to prevent	The nurses (home care professionals) conceptualised frailty in different ways; as an individual trait or as a result of the interaction between individual and environmental factors. Conceptualising frailty on a continuum between; as related to prevention and management (‘cure’) and as related to ageing as natural decline (‘care’). The nurses (home care professionals) considered the older peoples’ (service users’) different need and the care they had to provide**—**from minor tasks to extensive need [**Descriptive label:** Frailty and its relation to functional ability] The nurses (home care professionals) preferred to use other terms than frail or frailty, which they saw as more “professional” and specifying the individual’s needs, as they considered the former terms as too imprecise and including contrasting meanings [**Descriptive label:** Frailty and its ambiguity] The nurses (home care professionals) saw frailty as both potentially preventable through care/interventions such as physical activity, nutritional support and social support, while at the same time demonstrating that they acknowledged that it was not always preventable [**Descriptive label:** Frailty and its reversibility]	Notions—Justifications Notions—Uncertainties Implications—Nursing Practice
Wang et al. [61] United States of America	To describe HHC nurses’ understanding of and educational preparation for effective assessment of depression and frailty in older patients and to identify barriers to HHC nurses’ care related to the assessment and care management of depression and frailty in older patients	Home-based care Four home healthcare agencies in the great Nashville, TN area, USA. One private, nonprofit agency and three proprietary, for-profit agencies 10 RNs for interviews (range 25–64 years, Median = 53.5 years, ♀ = 9) 4 additional RNs for observations (age range 40–50 years, ♀ = 3) 16 older people observed (range 65 and 94 years, median 79 years, ♀ = 62%)	Qualitative pilot study Direct observations; N = 16 home visits Semi-structured interviews; N = 10 Qualitative content analysis (inductive-deductive), using an adapted SEIPS framework IBM SPSS: Descriptive quantitative analysis of data from observations **Categories** from interviews (SEIPS framework): Health care system; HHC agency; Technology and tools; HHC nurses; Home and community environment; Patient characteristics 14 themes in total under these categories	The nurses report a lack of education in frailty assessment, and few nurse visits incorporated such assessment, i.e., frailty was not routinely screened for in nursing practice. The nurses were familiar with the term frailty generally, still they were unsure of the exact meaning of the term. Despite a belief that screening for frailty fell within their job role they did not feel prepared to assess or manage frailty due to lack of education and training, this was referred to others; “Physical and occupational therapy [are better suited] because they are professionals…”. Assessment of frailty indicators that was done was primarily subjective (e.g., patient weight self-report), while objective measures were related to specific medical conditions. In interviews, assessment of (instrumental) activities of daily living were reported as prioritised, yet it was not noted during any of the observations. The findings might suggest that for both frailty and disability, what HHC nurses need is a higher-level understanding about how these deficits and symptoms interact and how these interactions lead to poor health outcomes. The participants reported a focus on ensuring safety, availability of equipment as well as availability and knowledge of informal caregivers. Having a recent history of falls, multiple chronic conditions, and physical exhaustion indicate elevated risk for frailty according to the participants. Barriers, which if addressed could facilitate nursing care delivery related to frailty included insufficient training and lack of standardised protocols/guidelines, documentation burden, limited reimbursement, difficulties getting insurance approval which affects the number of visits and type of equipment provided, lack of effective interdisciplinary collaboration and high caseload affecting the possibility to develop a trusting relationship important to gather important information	Nurses were unsure of the exact meaning of frailty and calls for more training is uttered [**Descriptive label:** Frailty and its ambiguity] Lack of knowledge (education and training) about frailty and its assessment, leads to frailty not being a part of the nursing practice [**Descriptive label:** Frailty and the lack of nursing knowledge] Described key-areas of importance when working with older people with frailty and risk factors for frailty [**Descriptive label:** Frailty and its “simple” description] The nursing practice was affected by several barriers, which if resolved might facilitate the nursing care [**Descriptive label:** Frailty and organisational challenges]	Notions—Uncertainties Notions—Deficiencies Notions—Justifications Implications—Nursing Practice
Archibald et al. [62] Australia	To understand how older people, including frail older persons in residential aged care, perceive and understand frailty	Facility- and home-based care Two aged care facilities (FBC) and one continued learning university (HBC) in South Australia. 39 older people (age range 62–99 years, mean 80.6 years, SD = 9.6) FBC; N = 17 HBC; N = 22 [sex not specified in numbers]	Interpretive descriptive qualitative design Focus groups interviews; N = 7 Thematic Analysis Theme: The old and frail: a static state near the end of life Theme: Frailty at any age: a disability model with the sub-theme; Perspectives of frailty as a dynamic state were common within the disability view Theme: Frailty as a loss of independence: control, actions and identity with the sub-themes; Frailty is seen as a loss of control over oneself and one’s environment and is closely tied to mobility; Frailty and a loss of independence is linked to identity and self-worth Additional theme describing important influencing factors (mediators) cutting across the three ways of describing frailty: Mediating factor: frailty is influenced by mental state and attitude, with the sub-themes; Within the ‘old and frail’ schema, mental state and attitude are seen as protective towards frailty but are entangled with choice and individualism; Mental frailty: attitude and mental state as a cause or type of frailty	Understandings of frailty varied significantly and despite the older people being familiar with the term frailty, it often lacked a specific meaning. Frailty was described according to three schemas for how older persons view frailty. First a model of frailty as old age, where frailty was related to the end of life and was largely unpreventable and unmodifiable. Second, a disability model where frailty was modifiable, could occur at any age and could affect isolated parts of the whole person (mostly described by the non-frail or prefrail participants living in community settings). Thirdly, an independence-focused model where frailty was seen as a static state, associated with age, loss of ability, control of one’s environment and oneself, loss of identity and self-worth. Mobility was central in this schema linked to independence, and mobility aids could be a sign of frailty. Aside from a disability model, views of frailty as unmodifiable permeated older persons’ perspectives. Still, the participants relating frailty to advanced age did generally acknowledge that not everyone becomes frail. Mindset, cognition, and emotions were discussed as important influencing factors cutting across the schema**—**entwined with attitude and choice and are indicated as mediators. Frailty was generally viewed negatively, often linked to end of life, and implicated with personal choice, specially related to mental frailty. Participants generally resisted self-identifying as frail and there was little correlation between the frailty assessments and participants self-identification as frail. Participants differentiated between different ‘types’ of frailty, i.e., physical, and mental frailty and discussed their relationship. Often physical frailty was perceived as “more real” and mental frailty was more associated with negative connotations	The older people understood frailty in a variety of ways and the term often lacked specific meaning [**Descriptive label:** Frailty and its ambiguity] Frailty equals negative connotations of being old, near the end of life and was associated with personal choice, especially related to mental frailty [**Descriptive label:** Frailty and its negative connotations] Frailty was primarily perceived as unpreventable and unmodifiable, but some still saw it as modifiable, occurring at any age and affecting isolated part of the person (mostly community-dwelling) [**Descriptive label:** Frailty and its reversibility] Frailty was a term the older people resisted identifying with them-self, even those assessed as frail [**Descriptive label:** Frailty and its discord] Described associated factors and mediators related to frailty [**Descriptive label:** Frailty and its “simple” description] Separating physical and mental frailty, with differing understanding of the relationship [**Descriptive label:** Frailty and its relation to functional ability]	Notions—Uncertainties Notions—(Pre)conceptions Notions—(Pre)conceptions Implications—Individual level Notions—Justifications Notions—Justifications
McGeorge [63] United Kingdom	To explore how mental health nurses construct and operationalize the concept of ‘age-related complexity’	Facility- and home-based care A large NHS mental health trust 13 RNs (♀ = 11) 5 working on wards 1 in care homes 1 in a general hospital 6 community psychiatric nurses [age not specified]	Constructivist grounded theory approach “Lightly structured” in-depth interviews Constant comparative method Category: Dynamic complexity. Themes: Components of complexity; Complexity as an abstract concept Theme (focus of this paper): The relationship between frailty and complexity with the sub-themes; Physical frailty versus multidomain complexity; Unidirectional frailty versus dynamic complexity; Decline versus recovery; Long-term conditions versus acute problems	Nurses in this study offered the consistent view that while frailty and complexity are related, they are neither mutually dependent nor mutually exclusive. Nurses saw the identification of frailty as straightforward or ‘obvious’. Frailty was exclusively used to describe physical states and attributes, while complexity is seen as a consequence of the interaction of needs across a number of areas. Unlike frailty, complexity is a dynamic state in which there can be movement back and forth, it emphasizes the possibility of improvement (becoming less complex) as needs are met or circumstances change	Frailty was exclusively related to physical states and attributes, and was believed to be obvious to identify [**Descriptive label:** Frailty and its relation to functional ability] Frailty was indirectly described as a static state with no possibility of transitioning between severity degrees [**Descriptive label:** Frailty, a constant process to defy, defeat conquer]	Notions—Justifications Notions—(Pre)conceptions
Schreuders et al. [64] England	To explore care home managers’ perspectives of the term frailty, how the care of residents living with frailty is managed and whether existing frailty guidelines are useful in the care home context	Facility-based care Seven care homes in the North of England. 8 care home managers: 5 previously worked as carers 2 were RNs 1 previously worked in hospitality management ♀ = 8 [age not specified]	Exploratory qualitative design Semi-structured interviews Thematic Analysis **Main themes:** Frailty is not specific enough; Providing individualised care to older people is more important than categorizing residents; Supporting residents to access outside support or expertise is a key role of the care home manager	The care home managers believed that the term frailty was not specific enough in a context where many are frail and individualised care is requisite, it was not useful in providing additional information to care management. The participants did not agree on the characteristics of frailty and the findings show that care home managers do not define the term frailty in the same way as in the medical literature. Frailty was described as related to a person’s physical ability but could also be relevant for people mental and cognitive decline or difficulties. The care home managers did not like to use the term frailty as it had negative connotations which they were afraid might be harmful to the person’s identity. It was seen as not in line with their responsibility of providing individually tailored care as labelling residents as ‘frail’ was incompatible with acknowledging them as individuals with unique needs. Furthermore, they did not believe identifying the residents as frail would facilitate access to outside support, even if they experienced barriers related to receiving such support. Care home managers valued a proactive approach and discussed how they used their knowledge and experience to recognise the need for, and arrange access to, outside expertise when caring for residents with frailty	The term frailty was believed to be too unspecific to be valuable in care, did not have an agreed set of characteristics nor did the understanding fit in line with medical literature [**Descriptive label:** Frailty and its ambiguity] Described frailty as mostly linked to physical ability and in some degree to mental or cognitive difficulties [**Descriptive label:** Frailty and its relation to functional ability] Care home manager use their knowledge and experience when managing care for older people with frailty [**Descriptive label:** Frailty and its intuitive nursing practice]	Notions—Uncertainties Notions—Justifications Notions—Deficiencies

^1^Adapted from Ritchey 1996

**Table 4:** Data extraction Q3_Models of Care

Author, year, country	Aim/research question(s)	Setting and participants	Design	Focus	Intervention components Models/Pathways/Guidelines and Outcome	Findings related to frailty or functional ability limitations
Strømme et al. [65] Norway	To describe the outcomes of a competence improvement programme (CIP) for the systematic observation of frail older patients in homecare *Research questions:* (1) How are the outcomes of a CIP in two homecare districts enacted by HCPs? (2) How do implementation and context influence the CIP outcomes?	Home-based care Two homecare districts in two municipalities in western Norway Observations: 21 HCPs 8 registered nurses 9 skilled health workers 4 assistants Focus groups: 15 HCPs 6 registered nurses 6 skilled health workers 3 assistants Semi-structured individual interviews: 5 managers 3 professional development nurses 1 assistant [age and sex not specified]	Qualitative mixed-method design (QUAL-qual) Participant observations (145 h) Focus group interviews; N = 5 Semi-structured individual interviews Qualitative content analysis **Five concepts** characterising the outcomes of the competence improvement programme: Frequency of vital sign measurements; Situational awareness; Expectations and coping level; Activities for sustained improvement; Organisational issues affecting CIP focus	Frailty- HCPs’ skills in recognising and responding to deteriorating frail older patients	The programme was multi-componential and consisted of a written compendium, a digital learning tool, a teaching day, and simulation-based training (including, the ABCDE algorithm and structured communication using ISBAR). An equipment bag, equipment backpacks, and a form to structure observation, decision-making, and communication were included in the programme	Substantial differences were revealed across the two homecare districts in how homecare professionals enacted new knowledge and routines resulting from the competence improvement programme. With one group showing positive changes, while the other showed little change. The differences were related to the frequency of vital sign measurements, coping levels, and situational awareness, in which successful outcomes were shaped by implementation issues and contextual setting. This involved whether routines and planned activities were set to follow up the improvement programme, or whether organisational issues such as leadership focus, resources, and workforce stability supported the programme. Several HCPs in the group with little change considered the need for measuring in homecare as redundant
Islam et al. [66] Norway	To investigate the impact of introducing a specific model of integrated care for frail elderly patients, the Holistic Continuity of Patient Care (HCPC) programme, specifically whether the HCPC programme contributes to improved health and well-being, experience of care and resource utilisation	Home-based care N = 209 older people (mean of mean = 81.73 years old) *IG:* 120 older people (mean = 79.87 years, SD = 9.924♀ = 99.63) *CG:* 89 older people (mean = 83.59 years, SD = 7.831 ♀ = 99.449%)	A quasi-experimental design and linear mixed methods, and conducts a multi-criteria decision analysis (MCDA)	Functional ability and improving patient pathways for older people with frailty	The Integrated care programme focuses on functional ability rather than on disease and impairment. There were three core differences between the HCPC programme and usual care. First, *the initial and follow-up* (6 weeks) assessment of the patient’s level of functioning by validated tools, second, t*he “everyday rehabilitation”* informed by the patient’s own goals for activities of daily living, third, t*he early involvement of the patient’s GP*, within 2 weeks after enrolment. Additionally, a *new professional role* was also developed as part of the programme; a designated primary contact (coordinator) working in the municipal care service, notably a nurse or a social worker, responsible for individual patient follow up	The results showed that older patients enrolled in the HCPC program experienced better outcomes compared to those receiving usual care in the municipalities. The MCDA results indicated that HCPC was preferred to usual care irrespective of stakeholders (patients, partners, professionals, policy makers and payers). The better performance of HCPC was mostly driven by improvements in enjoyment of life, psychological wellbeing, and social relationships and participation. Results also reflect that involving more health personnel in HCPC may provide better care but at the cost of more decision-making being left to the professional care providers, which can negatively affect the patients’ feeling of autonomy
Lewis et al. [67] Ireland	To examine the effect of an established Community Virtual Wards (CVW) on pre-defined health trajectories (between “stable”, “deteriorating”, and “unstable” states) and characteristics that increased the likelihood of adverse healthcare outcomes (hospitalization, institutionalisation and death)	Home-based care (One Community Virtual Ward in a single centre in Ireland) 88 older people (mean = 82.8 years, SD = 6.4, ♀ = 58)	Non-experimental correlational design using prospective data over a period of 90-day postadmission to the CVW	Delaying or reversing frailty. Supporting older people to remain at home and transitioning from hospital to community	The model of care supported older people to remain at home and transitioning from hospital to community. Care was coordinated by a senior nurse working with other healthcare professionals both in primary and secondary care. The model operated under three levels of CVWs separated to include red (high risk) amber (moderate risk) and green (low risk). The intervention started with a triage phase where home assessment and prioritisation of care needs was done by the senior nurse. Thereafter, the older people were admitted into either the Red (high risk) CVW or the Amber (moderate risk) CVW. Interval assessments were done, and patients transferred to the different levels accordingly	The results show that a CVW model can provide a framework for monitoring and case management to support older people to remain at home or identify those at risk of institutional care. The model has the potential to support a frail older population at home delaying and/or reversing the downward trajectories of frailty. The use of defined health states assisted to stratify those at lower or higher risk. Achieving stability within 30 days and remaining stable at 60 days were associated to remaining at home
Galik et al. [68] United States of America	The purpose of this study was to test the impact (effectiveness) of Function-Focused Care for the Cognitively Impaired Intervention on nursing home residents with dementia and the nursing assistants who care for them	Facility-based care N = 180 103 older people with cognitive impairment (mean = 83.7 years, SD = 9.9, ♀ = 79.77%) 77 nursing assistants (mean = 41.60 years, SD = 12.8, ♀ = 96%)	6-month cluster-randomized controlled trial using repeated measures	To support NH staff to actively engaging cognitively impaired residents in functional and physical activities that are person centred	The FFC-CI intervention included four components. First, environment and policy/procedure assessments, including evaluation of the environment and nursing home policy and procedures to determine whether they presented barriers to implementation of a FFC approach. Second, education, including education of nursing home staff and families about FFC (thirty-minute in-service + handouts). Third, developing function focused goals, including person-centred individual resident function and physical activity goals which was initiated through assessment and discussions with the FFC nurse, resident, family, staff, and facility champions. Fourth, mentoring and motivating, including ongoing education and motivation of staff by FFC nurse and facility champions (selected staff)	There were significant improvements in the amount and intensity of physical activity (by survey and actigraphy) and some improvement was seen in physical function in the treatment group. In addition, they were less likely to fall. Nursing assistants were also observed to be providing a greater percentage of function focused care during resident care interactions in the treatment group at 6 months following the completion of baseline measures (even if this was a small, significant increase (63%–66%))
Henskens et al. [69] the Netherlands	To evaluate the effects of three movement stimulating interventions on QoL and ADL performance in NH residents with dementia	Facility-based care N = 87 older people with dementia (Age range = 71–100 years, mean (SD) in the four groups = 86.95 (7.21), 86.05 (5.86), 85.14 (4.64), 84.73 (4.55), ♀ = 67) Mean of mean = 85.7 years old	6-month double parallel randomised controlled trial	Activities of daily living (functional ability/limitations) and quality of life	The intervention was separated into three groups: ADL training alone, a multicomponent exercise training alone, and a combined ADL and exercise training. In the individually based ADL training intervention, nursing staff were asked to stimulate movement during daily care tasks by encouraging residents to perform as much of their self-care as independently as possible throughout the day. The multicomponent exercise training intervention consisted of strength and aerobic exercises, three times per week, for 30–45 minutes per sessions, guided by qualified movement teachers. For each ward per ADL location, ambassadors, including two nursing staff, received three 3-hour educational sessions by qualified physio- and occupational therapists. These individuals were then responsible for sharing their knowledge with the other nursing staff	No effects were found of the three movement interventions on ADL performance. Although no effects were found of ADL training on ADL performance, an observed trend showed a maintenance in ADL performance in the ADL group, and a decline in the care-as-usual group. Although these differences were not significant, maintenance in ADL is considered a positive finding, as NH residents with dementia typically experience a decline in ADL performance
Kerse et al. [70] New Zealand	To assess the effectiveness of an activity programme in improving function, quality of life, and falls in older people in residential care	Facility-based care N = 682 older people (mean = 84,3 years, SD = 7,2, ♀ = 502) *IG* = 330 (mean = 84.4 years, SD = 7.2, ♀ = 240) *CG* = 352 (mean = 84.1 years, SD = 7.2, ♀ = 262)	(Pragmatic) cluster randomised controlled trial with one year follow-up	Function, quality of life and falls *Note:* In seven of the 41 homes, the assessor was unblinded at some time during follow-up. This potentially affected measures on 56 activity participants and 41 social participants	The intervention group were offered a goal setting and individualised activities of daily living activity programme by a gerontology nurse, reinforced by usual healthcare assistants. There was a focus on imbedding the activities in the daily activities. The intervention included: goal setting, functional assessment and activity programme design, staff implementation (training of healthcare assistants) and ongoing support. In the control group the residents received usual care and social visits	The goal-oriented programme based on activities of daily living had no impact overall. However, in contrast to residents with impaired cognition, those with normal cognition in the intervention group may have maintained overall function and lower limb function. Still no changes occurred in observed function, quality of life, or falls. Neither achievement of goals nor compliance made any difference to improvement in function. Residents with impaired cognition showed no maintenance of function and the likelihood of depression increased in the intervention group. No other outcomes differed between groups

The majority of the included papers were quality assessed by the first author (IRF), whereas the second author (ERG) and last author (GB) independently and randomly supported the assessment. Our quality assessment aimed to identify gaps in the literature related to high-quality research and determine areas not requiring further investigations [cf. 71]. The Critical Appraisal Skills Programme (CASP) checklist for qualitative [72] and randomised controlled trials [73] was used. The appraisal tool for cross-sectional studies (AXIS) [74] was used for cross-sectional designs, and the Johanna Briggs Institute Critical Appraisal Tool was used for quasi-experimental designs [75]. The ethical quality of the papers was assessed as recommended by Weingarten et al. [76] and inspired by Westerdahl et al. [77] (Table 5). We chose our appraisal tools based on their common usage in health service research and the high level of familiarity they offer. None of the critical appraisal tools recommended the use of scores. However, to be able to offer a clear map of the paper’s quality and its ethical considerations we decided to calculate the number of ‘yes answers’ for each tool (Table 5). The total number of ‘yes’ answers was then divided into quartiles (q), where q1 and q2 represented low quality, q3 represented medium quality and everything above q3 represented high quality. Our approach felt acceptable as no papers were excluded and no evidence was weighted based on these procedures.

**Table 5:** Patterning chart: Design and critical and ethical appraisal Q1–Q3

	Question			Country	Context		Population				Design				Quality appraisal					
Author(s) and year	*Q1*^*1*^ *(N* *=* *5)*	*Q2*^*2*^ (*N* *=* *15)*	*Q3*^*3*^ *(N* *=* *6)*		Home- Based Care	Facility- Based care	Registered Nurses (N = 66)	Non-registered Nurses (N = 190)	Older people (N = 1689)	Significant others (N = 16)	Descriptive Qualitative Design^4^ (N = 14)	Classic Qualitative Design^5^ (N = 6)	Non-Experimental Design (N = 3)	Experimental Design (N = 3)	Critical appraisal			Ethical considerations		
															High	Medium	Low	High	Medium	Low
Imaginário et al. [45]	✘			Portugal		✘			✘				✘			✘		✘		
Lehto-Niskala et al. [46]	✘			Finland		✘				✘	✘				✘			✘		
Letho et al. [47]	✘			Finland		✘	✘	✘	✘		✘				✘			✘		
Palacios-Ceña et al. [48]	✘			Spain		✘			✘			✘			✘			✘		
Sacco-Peterson and Borell [49]	✘			Sweden		✘	✘	✘	✘			✘			✘			✘		
Bjerkmo et al. [50]		✘		Norway	✘				✘		✘				✘			✘		
Britton [51]		✘		England	✘		✘				✘				✘				✘	
Lloyd et al. [52]		✘		UK	✘				✘		✘				✘			✘		
Nicholson et al. [53]		✘		UK	✘				✘		✘				✘				✘	
Nicholson et al. [54]		✘		UK	✘				✘		✘				✘			✘		
Obbia et al. [55]		✘		Italy	✘		✘	✘				✘			✘			✘		
Papadopoulou et al. [56]		✘		Scotland	✘		✘	✘**			✘				✘				✘	
Skilbeck et al. [57]		✘		England	✘				✘			✘			✘			✘		
Strømme et al. [58]		✘		Norway	✘		✘	✘**			✘				✘			✘		
Søvde et al. [59]		✘		Norway	✘				✘			✘			✘				✘	
Voie et al. [60]*		✘		Norway	✘		✘	✘**			✘				✘			✘		
Wang et al. [61]		✘		USA	✘		✘				✘				✘					✘
Archibald et al. [62]		✘		Australia	✘	✘			✘		✘				✘			✘		
McGeorge [63]		✘		UK	✘	✘	✘					✘			✘				✘	
Schreuders et al. [64]		✘		England		✘	✘	✘			✘				✘			✘		
Strømme et al. [65]*			✘	Norway	✘		✘	✘			✘				✘			✘		
Islam et al. [66]			✘	Norway	✘				✘				✘				✘	✘		
Lewis et al. [67]			✘	Ireland	✘				✘				✘			✘		✘		
Galik et al. [68]			✘	USA		✘		✘	✘					✘	✘			✘		
Henskens et al. [69]			✘	Netherlands		✘			✘					✘		✘		✘		
Kerse et al. [70]			✘	New Zealand		✘			✘					✘	✘				✘	

Q1: Functional ability limitations, 2 Q2: Frailty, 3 Q3: Models of Care, 4 Sandelowski [78], 5 Cresswell (2007), *From the updated search, ** The paper does not separate registered- and nonregistered nurses, hence included as the latter

### Stage 5: collating, summarising and reporting the results

In this stage our choice of summarising via a descriptive approach appeared to be the most appropriate. Thus, we utilised concepts such as coding and categories whilst excluding the final stage of abstraction as described in most recommended approach**—**content analysis [79, 80]. Our rationale was twofold: (i) the concept of ‘summarising’ [26, 44, 71, 79] are still enigmatically described, (ii) Morse’s [81] definition of a theme (sc. thematic analysis) as the ‘essence’ that runs through the data (a red thread) and of a category (sc. content analysis) as a set of similar data that have been sorted together. Despite choosing the latter we have opted to not label our procedure as thematic- nor as content analysis. Instead, we offer a thorough presentation of our process of analysis below.

Our process began with a careful reading of the findings of the included papers by the first author (IRF). The pertinent text that was evaluated to answer our questions was extracted (Column 5, Findings) into the extraction sheet (Tables 2 and 3), and the text was then broken down into smaller parts, making it possible to inspect and understand the individual parts while searching for patterns in the data, i.e., analysis [82]. In the third phase, the sorting and categorisations [81] of all the acknowledged patterns were given an identifier, that is, a tentative descriptive label that resulted in 17 descriptive labels (Column 6, Sorting of findings). Next, in phase four, to create an overall pattern of our sorting of the text excerpt, a patterning chart (Table 6) for Q1 and Q2 was created. This phase was inspired by Waigwa et al. [83] and the visualisation of their findings. Our work with the patterning chart resulted in the identification of constructs representing two categories, ‘implications from the states’ for nursing practice, everyday life and the older individual and several descriptive and experiential ‘notions of the state’, such as justifications, (pre)conceptions, uncertainties and deficiencies. The patterning chart composed, supported the visualisation of the findings to gain a logical structure of the narrative results. The whole process was characterised by an iterative process going back and forth between the parts of the text. In these phases, the last author (GB) randomly inspected the thoroughness and relevance of the work conducted on a regular basis. Team meetings were held regularly to ensure the rigour of the process. For Q3, the extracted data were shortened and compiled into brief narrative descriptions (Table 4).

## Findings

Our search resulted in 18,875 potentially relevant records after the removal of duplicates. After title–abstract screening, 76 papers were read in full text, resulting in 26 papers being included and 50 excluded; approximately 68% of them were assessed as not answering any of our questions, whereas the remaining were assessed as not meeting our inclusion criteria.

### Descriptive findings

Twenty-six papers were evaluated to answer our questions. Q1 and Q2: How are the conditions of functional ability limitations and frailty among older people in home- or facility-based care described by key stakeholders? Q3: What models of care (nursing activities and/or interventions) can be identified as targeting these two conditions? The majority (77%) answered our first two questions. Of these, 19% covered functional ability limitations, and 58% covered frailty. Moreover, 23% of the papers covered MoCs targeting these two conditions. Four focused on functional ability limitations, and two focused on frailty. The papers represented research from Nordic countries (n = 9), Europe (n = 13), North America (n = 2) or Oceania (n = 2) (Table 5).

A total of 1961 participants were represented. Older people represented 86% (1689) of the total number of participants. Registered or nonregistered nurses represented 3.3% and 9.7%, respectively, while 1% represented significant others. Included papers answering Q1 represented research conducted in facility-based care (100%), whereas 80% of the papers answering Q2 represented research conducted in home-based care. One paper answering Q2 was set in facility-based care (6.7%), whereas two were set in both home- and facility-based care (13.3%). Papers answering Q3 were equally set between home- and facility-based care (50–50%). The majority of included papers (54%) were conducted with a descriptive qualitative design [78], and 23% were conducted with a classical qualitative design [84]. The remaining 23% were conducted with a quantitative design, where 11.5% of them utilised an experimental design and 11.5% used a nonexperimental design (Table 5).

### Descriptions of functional ability limitations

Five papers representing facility-based care [45–49] answered Q1. Two accounted for the perspective of older people alone [45, 48], whilst two accounted for both older people and nurses’ perspectives [47, 49]. One paper accounted for the perspective of significant others [46] (Table 6).

**Table 6:** Patterning chart: Categories for Q1–Q2

	State		Context		Perspective			Implications from the states^1^			Notions of the states^1^			
*Author(s) and year*	*Q1*^*2*^	*Q2*^*3*^	*Home-* *based Care*	*Facility-based care*	*Nurses*	*Older people*	*Significant others*	*Nursing practice*	*Everyday life*	*Individual* *level*	*Justifications*	*(Pre)conceptions*	*Uncertainties*	*Deficiencies*
Imaginário et al. [45]	✘			✘		✘		✘	✘		✘			
Lehto-Niskala et al. [46]	✘			✘			✘	✘	✘			✘		
Letho et al. [47]	✘			✘	✘	✘			✘	✘	✘	✘		
Palacios-Ceña et al. [48]	✘			✘		✘					✘	✘		
Sacco-Peterson and Borell [49]	✘			✘	✘	✘		✘	✘	✘				
Bjerkmo et al. [50]		✘	✘			✘			✘		✘			
Britton [51]		✘	✘		✘			✘			✘	✘	✘	✘
Lloyd et al. [52]		✘	✘			✘			✘	✘	✘			
Nicholson et al. [53]		✘	✘			✘			✘	✘				
Nicholson et al. [54]		✘	✘			✘			✘	✘				
Obbia et al. [55]		✘	✘		✘			✘		✘	✘	✘	✘	✘
Papadopoulou et al. [56]		✘	✘		✘			✘				✘		✘
Skilbeck et al. [57]		✘	✘			✘			✘					
Strømme et al. [58]		✘	✘		✘			✘						✘
Søvde et al. [59]		✘	✘			✘			✘					
Voie et al. [60]*		✘	✘		✘			✘			✘		✘	
Wang et al. [61]		✘	✘		✘			✘			✘		✘	✘
Archibald et al. [62]		✘	✘	✘		✘				✘	✘	✘	✘	
McGeorge [63]		✘	✘	✘	✘						✘	✘		
Schreuders et al. [64]		✘		✘	✘						✘		✘	

^1^As described by the participants in this scoping review, ^2^Q1: Functional ability limitations, ^3^Q2: Frailty, *From the updated search

#### Implications from the state of functional ability limitations

The descriptive category ‘implications from the state of functional ability limitations’ mapped out descriptions of the consequences for (i) the nursing practice given and on offer, (ii) the older people’s everyday life activities and (iii) for them as individuals (Table 6).

Descriptions of the consequences of functional ability limitations for nursing practice were identified from the perspectives of all three key stakeholders in facility-based care [45, 46, 49]. Nurses’ ‘well-intended’ actions related to functional abilities affected the care that older people received [45, 49]. Regardless of older people’s functional level or degree of autonomy, nurses were perceived as replacing older people in self-care tasks [45]. When the nurses did not assess their support and actions related to activities daily life (ADLs), interventions intended to facilitate these tasks for the older people could become ineffective [49]. Despite the nurse’s close bedside presence, older people could experience more difficulties and need more assistance than the nurses realised [49]. Nursing practice was described as being affected by contextual and organisational challenges [46, 49]. For example, significant others described organisational challenges, such as a lack of resources, highlighting the ambiguity but also the necessity of their involvement and role in supporting the older person’s functional ability next to the nursing staff in their practice [46]. Contextual difficulties in nursing practice, such as the lack of formalised care plans and being chronically short staffed, were described as forcing nursing staff to work in ways contrary to what they meant was best practice. For example, replacing toileting care with the prescription of diapers, even though the older person was continent, thus increasing the risk of harm to the older person’s self-care abilities and dignity [49].

The implications of functional ability limitations for everyday life include one aspect highlighted by both older people [45–47] and significant others [46]: the importance of focusing on more than merely ADLs, or ‘daily chores and independence, when considering the state. Both emphasise the importance of including pleasurable activities, social relations, emotional needs and preferences in everyday life [45–47]. The specific context and routines inherent to the nursing home environment made maintaining autonomy and dignity related to ADLs more challenging for the older people [49]. Functional ability limitations were described as having consequences not only for older people’s everyday lives but also for them on an individual level [47, 49]. Although valuing participation, they described their struggles in doing so because of their functional ability limitations [49] as well as their struggle to withhold their independence [47]. The later was described as an important part in easing the burden for the already pressured nursing staff [47].

#### Notions of the state of functional ability limitations

The descriptive category ‘notions of the state of functional ability limitations’ mapped out several descriptive and experiential notions of the state, such as justifications and (pre)conceptions (Table 6).

The notion of justifications for functional ability limitations was explained mainly as being related to concepts such as capacity and independence in ADL [45, 47]. The nurses explained functional ability limitations related to ADLs as being related to activities of daily living—being dependent or independent. Alternatively, they saw it as an abstract term that included physical, social, and psychological domains, for example, standardised indicators [47]. Older people’s descriptions focused more on the different ways of coping with functional difficulties rather than on a dichotomy of independence or dependency. They explained it as a more versatile concept, including ADLs, but ranging from needing help and coping with difficulties to feeling like a burden [47]. On the other hand, explanations could also entail notions about being ‘noncapable’ without this being related to needing support in daily activities or the use of assistive devices. The older people resisted showing any signs of, for example, being incapable of walking because this was seen as a potential first sign of dependence [48].

(Pre)conceptions concerning the states described how older people struggled to resist the label of being dependent, for example, being solely a ‘care-receiver’ or being ‘noncapable’ [47, 48]. Even if they did have functional limitations, they worked hard to avoid being labelled as such because this could risk them being socially excluded and being stuck with the label permanently. Negative connotations, or discrimination against those labelled ‘noncapable’, were reflected in how the nursing staff acted [48]. (Pre)conceptions were also described concerning the power or influence that older people had on functional ability limitations themselves, with both older people and significant others believing that older people could influence their functional abilities to some extent [46, 47].

### Descriptions of frailty

Fifteen papers [50–64] answered Q2. Twelve was conducted in home-based care [50–61], two in both contexts [62, 63] and the last one in facility-based care alone [64]. Seven of them reflected the perspective of older people [50, 52–54, 57, 59, 61], and the remaining eight reflected the perspective of nurses [51, 55, 56, 58, 60, 61, 63, 64] (Table 6).

#### Implications from the state of frailty

In the descriptive category ‘implications from the state of frailty’, consequences for nursing practice, for older people’s activities of everyday life and for them as individuals were mapped out (Table 6).

The implications from frailty for nursing practice were accounted for by the nurses, who described that their practice regarding the identification, detection and early recognition of frailty was predominantly guided by their ‘intuition’ [51, 56, 58]. The nonspecific nature of signs and symptoms of frailty was described as hindering their practice of early recognition resulting in a sense of concern while also demanding substantial knowledge of the older person [58]. They also described an experience-based reactive practice concerning the identification and detection of frailty [56]. Several organisational challenges were described to negatively influence their nursing practice related to frailty [51, 58, 61]. Time pressure, lack of staffing, gaps in service, instruments available for identification of frailty not adapted to the setting [51], lack of standardised tools, lack of training and interdisciplinary collaborations [61] and task-oriented services [58] were described. The prevention of frailty was feasible [55, 60], but early detection and effective preventative interventions were described as requiring a systematic reconstruction of the organisation [55]. Interventions such as physical activity, nutritional support and social support were exemplified to potentially prevent frailty.

The implications of frailty for older people’s activities of everyday life include descriptions of how physical challenges, along with contextual changes, and the physical and social environment limited older people’s everyday lives. However, despite challenges, most older people wanted to live at home while expecting to have to move into facility-based care at some point in time [50]. The descriptions of a constant daily struggle to sustain and develop routines aimed at maintaining their identity as capable and independent were also identified. These struggles disrupted and restricted their everyday life while demanding varying degrees of adaptation to losses, difficulties and the physical and psychosocial changes they experienced. Frailty was described as a dynamic and persistent state of imbalance [50, 53, 54] and as a continuing downwards spiral from which individuals could not escape, causing experiences of fear, anxiety, uncertainty and a sense of a meaningless everyday life [52, 57, 59]. Frailty was also described to have an incremental effect on older people as individuals, especially on their self-image and self-worth [52–55, 62]. Despite being assessed as frail by nursing staff, older people did not identify with this concept or wish to be labelled as such, nor did they desire to be classified as needing help [53–55, 62].

#### Notions of the state of frailty

In the descriptive category ‘notions of the state of frailty’, constructs concerning a variety of justifications, (pre)conceptions, uncertainties and deficiencies about frailty were mapped out (Table 6).

The descriptive justifications related to frailty included the nurses’ rather straightforward accounts of frailty. The nurses described risk factors for frailty such as loneliness, cognitive problems, psychological distress, recent falls, psychological exhaustion and the need for mobility- or other assistance equipment, whereas having an adequate social network was described as a protective factor [51, 55, 61]. An assessment of (instrumental) ADLs was prioritised over frailty in practice, and the meaning of frailty was described by some nurses as unclear [61]. Others explained frailty as both an individual trait and interaction between individual and environmental factors. The nurses’ explanations related to the care they provided entailed descriptions of frailty on a continuum from preventative care and management, to related to ageing and a natural decline [60]. Frailty was also explained mainly as a physical state and related to physical ability [63, 64]. Some nurses explained that, in relation to their understanding of age-related complexity, the identification of the state of frailty among older people was obvious [63]. Older people explained frailty as either two separate entities, physical and mental frailty, or as one entity, but their descriptions of the possible relationships between these two types of frailty differed [50, 62]. Physical frailty was explained as more ‘real’, whereas mental frailty was related to mainly negative connotations. They described mindset, cognition and emotions as factors affecting frailty while relating frailty to loss of ability, independence (mobility) and loss of control over one’s life [62].

On the other hand, some of the older peoples’ justifications included more nuanced descriptions. Some described frailty not exclusively as an individual trait or condition but rather as an interplay between age, health-related changes, contextual challenges and the physical and social environments [50]. Frailty was also described as the following distinct patterns: stability, unbalancing and overwhelming, reflecting how the person adapts to increasing difficulties and losses and reintegrating their sense of self into a cohesive narrative [52]. The latter also entailed descriptions of social losses, depressive symptoms, anxiety and existential suffering, which could culminate in a tipping point where the older person surrendered [52]. Frailty was described as being connected to increasing age to different degrees by older people. Some adapted to these changes, whereas others found it difficult to accept [50].

Descriptions about (pre)conceptions concerning frailty represented descriptions related negative connotations, such as being old, needing help and/or being vulnerable, by both nurses and older people [51, 55, 56, 62]. Some even related personal choice to frailty, especially mental frailty [62]. Frailty could be seen as a static state that is unpreventable and unmodifiable [62, 63]. Simultaneously, frailty was also described, mainly by non-frail or prefrail community-dwelling older people, as modifiable regardless of age and as potentially affecting isolated parts of the person [62].

Descriptions by both nurses and older people of uncertainties related to frailty reflected a term lacking a specific meaning and uncertainties related to its definition [51, 55, 60–62, 64]. Some nurses could not distinguish between frailty, disability and multimorbidity [55]. Frailty was even seen as too unspecific to be valuable in practice working with this complex population [64], or the nurses preferred to use a more ‘professional’ term focusing on the older person’s needs [60]. Descriptions of deficiencies were identified, and the lack of knowledge related to frailty and its assessment and detection was described among nurses [51, 55, 56, 58, 61]. This lack of knowledge could, in some circumstances, result in less awareness of frailty in nursing practice [61].

### Identified models of care and/or nursing interventions

Six papers were identified answering Q3 that is; what models of care (nursing activities and/or interventions) targeting functional ability limitations and/or frailty in relation to older people can be identified, where three were conducted in home-based care [65–67] and three in facility-based care [68–70]. Four papers focused mainly on older people [66, 67, 69, 70], one focused on both older people and nurses [68], and the last focused on nurses [65]. The latter represented research with a qualitative descriptive design, whereas three used an experimental design [68–70] and two a nonexperimental design [66, 67]. Furthermore, four targeted functional ability limitations [66, 68–70], and the two remaining targeted frailty [65, 67]. Four included educational interventions targeting nursing staff (Table 4).

In one of the papers conducted with a descriptive qualitative design, the intervention focused on increasing the nurses’ skills in recognising and responding to deterioration [65]. This paper revealed an alterable effect of the intervention as its delivery was described to be affected by implementation and contextual issues [65] (Table 4). Moreover, three papers presented research where different types of care models were implemented: (i) integrated care [66], (ii) a community virtual ward [67] and (iii) function-focused care [68]. All the MoCs implied positive result but without statistically significant effect related to the implemented and tested MoCs. The integrated care model [66] showed that the intervention was preferred over usual care. The difference in preference was especially related to the outcome measures; enjoyment of life, psychological well-being and social relationships and participation, while the effect on physical functioning was less pronounced. The community virtual ward [67] implied the potential to support older people in remaining at home as well as to delay or reverse the downwards trajectory of frailty, whereas the function-focused care model [68] showed increased physical activity and some improvement in physical functioning. Additionally, the latter paper indicated, a small statistical change, that nursing assistants provided more function-focused care [69]. Finally, two papers focused on interventions consisting of different forms of ADL training, but neither of these interventions had a statistically significant positive effect on ADLs [69, 70]. Both papers showed that ADLs were maintained in the intervention group, except for participants with cognitive impairment [70].

### Descriptive findings from the critical and ethical appraisal

Critical and ethical appraisals were conducted (Table 5). All the papers with a qualitative design were assessed to be of a high quality [46–65]. However, only 30% of the papers addressed, to some extent, the relationship between the researcher and participants [47, 49, 57, 59, 61, 65]. Among the papers with a nonexperimental design, 67% were of a medium quality [45, 67], whereas 33% were of a low quality [66]. Two randomised controlled trials were of a high quality [68, 70], and one was of a medium quality [69]. Regarding ethical appraisal, 73% of the papers were assessed to be of a high ethical quality [45–50, 52, 54, 55, 57, 58, 60, 62, 64–69], whereas 23% were of a medium [51, 53, 56, 59, 63, 70] and 4% of a low quality [61]. Only 15% of the papers addressed the handling, storing or protection of data beyond simply stating following guidelines [58, 60, 65, 66].

## Discussion

This scoping review mapped the literature on three questions: Q1 and Q2: How is the condition of functional ability limitations, and the condition of frailty among older people in home- or facility-based care described by the key stakeholders? Q3: What models of care can be identified as targeting these two conditions in relation to older people in home- or facility-based care? The following discussion is presented within the framework of PAGER: patterns, advances, gaps, evidence for practice and research recommendations [27].

### Patterns

Our findings could be plotted into two categories, that is, implications and notions of the states, creating a detectable pattern (Table 6). The pattern primarily represented older people in facility-based care (75.8%). While the nurses, being in minority (13%), mainly represented the home-based care context. Our pattern revealed that the findings largely represented a Central and Northern European perspective (Table 5). The states of functional ability limitations (Q1) and frailty (Q2) were described slightly differently among the key stakeholders. The findings highlighted a discrepancy between the older people’s subjective, existential perspective and the nurses’ more objective, ‘matter-of-fact’ perspective, particularly regarding frailty as a physical state related to physical ability. The older people described the states of functional ability limitations and frailty in terms of identity loss and an emotional struggle to remain independent, challenging the stereotypical views of dependency associated with ageing. Negative connotations or ‘labels’ related to both states as being old, vulnerable and dependent [85] were rejected by the older people (Table 6). The importance of positive connotations was described in relation to older people’s efforts to adapt and accommodate their situation related to both states (Table 6). Two published meta-syntheses corroborate our findings regarding the state of frailty and the older people’s efforts to challenge this state and its negative connotations [86, 87]. Nursing practice targeting functional ability limitations and frailty were predominantly described as being reactive, which was based on nurses’ experiences and guided by their ‘intuition’ rather than as encompassing structured activities guided by MoCs. Surprisingly, the majority of the few MoCs identified (Q3) addressed functional ability limitations, even though the latter has seemed to attract relatively little attention within research into nursing compared with the state of frailty.

### Advances

Our findings seem to imply some clearly detectable advances. The findings can be positioned in the frame of the core principles of nursing, here being seen as a nursing practice departing from a humanistic and holistic perspective [cf. 88]. To the best of our knowledge, this is the first published scoping review mapping descriptions about both the state of functional ability limitations and frailty in home- and facility-based care from the perspective of nurses, older people and significant others. The same seems true for MoCs aiming to support nursing practice targeting these states. Therefore, it is notable that our findings propose a clear advancement in that the included papers explored older peoples’ perspectives of frailty in home-based care as we sparsely identified the same substantial acknowledgment in relation to the state of functional ability limitations. The descriptions from older people highlight the importance of seeing the individual in a way that is consistent with the humanistic and holistic perspective of nursing and promoting independence [cf. 1, cf. 88] rather than focusing on older people’s individual health conditions. Furthermore, challenging stereotypical views of ageing [85] seems especially relevant for strengthening older people’s self-efficacy because it is estimated that the proportion of older people living with frailty both nationally and internationally will increase substantially [cf. 89]. Despite these advancements, the perspective of older people still needs to be strengthened in the dominate understanding of frailty today. However, the same advancements cannot be said to exist related to nurses’ and significant others’ perspectives nor for descriptions of functional ability limitations. Neither has the same advancements been identified related to MoCs.

### Gaps

Our findings suggest that the research into nursing may not have fully explored the state of functional ability *per se* or, as here, functional ability limitations among older people. A clear gap was that we did not identify similar findings for functional ability limitations as for frailty regarding notions about uncertainties or deficiencies. Instead, the descriptions and experientials seemed to justify and conceive of functional ability limitations as a ‘natural’ and intrinsic part of ‘normal ageing’ (Table 6), representing a somewhat ‘outdated’ nursing practice. Viewing functional limitations as a normal part of ageing or still using concepts such as being dependent or independent in the care of older people is, in our view, counterproductive. By adopting new perspectives on functional ability limitations and responding to the cues in older people’s descriptions, which portray a deliberate ‘fight’ against these limitations and negative labelling, we can shift the scope of nursing practice into increasing proactive thinking, functions and working modes. Instead of merely compensating for limitations, we can develop nursing interventions that emphasise older people’s ability to strive, adapt, accommodate and cope. Thus, focusing on older people’s functional abilities instead of limitations could push the here earlier described reactive nursing practice towards a more proactive and preventative nursing practice for older people in these two contexts. The latter should be prioritised as we know that the home-based care context is the fastest growing health service area for older people both in Nordic countries and Europe [4, 6]. It is here that nurses and—a relevant nursing practice—have favourable possibilities for making an impact. Despite the importance of advancing this field, this review also identified gaps related to the perspective of nurses and a lack of MoCs. Research has shown that frailty is preventable and reversible [24] and, thus, associated negative health outcomes [13]. However, early detection is vital, highlighting the need to support nurses in working effectively and systematically, especially in the demanding context of home-based care. Therefore, the lack of MoCs here is concerning.

### Research recommendations

Regarding research recommendations, our findings, quality and ethical appraisal evaluations included, coupled with the recent influx of literature reviews in this field [86, 87, 90], have shown that we seem to have reached a point of saturation on the subject of older people’s descriptions of frailty in home-based care. Hence, it is safe to propose that this does not require any further exploration through qualitative research. Particularly as the majority of the included papers here clearly reflected high quality execution in the quality and ethical appraisal. Rather, what should now be prioritised is using this existing knowledge to develop and test feasible solutions to increasing early detection and prevention of frailty through (non)experimental designs. However, the latter warrants some caution regarding their methodological stance as the majority of those included in this review reflected a medium quality and, a total of 33% of them reflected low quality. Regarding the descriptions concerning functional ability, MoCs or research focusing on nurses’ perspectives related to either state our findings clearly implies the need of more well conducted studies regardless of whether the research questions imply a qualitative or quantitative design.

Regarding the state of frailty, our work further highlights the importance of embracing the multidimensional approach to frailty. The ongoing debate among academics and clinicians about the uni- and multidimensional approaches, coupled with the absence of a clear and comprehensive definition of frailty [91], has not significantly advanced the field. In addition, there is controversy over whether frailty should be considered a precursor to functional ability limitations or whether it should be considered a part of frailty (12). This suggests that, to make progress in the field, it may be necessary to address the conceptual confusion surrounding these two states, especially within research into nursing. Several researchers have advocated for a multidimensional approach to frailty, emphasising overall functioning and the potential reversibility and preventability of these states [92–94]. Furthermore, research implies a shift within the nursing profession where the perspective of nursing and nursing practice in relation to the state of frailty is more prominent [95–97]. This shift is vital because others [95, 96] have suggested that nurses in community care are exceptionally situated to facilitate assessments and management of the ever-changing phenomenon of the state of frailty in the care of older people. Considering this, we propose that it is possible that the two states of interest in this review must be explored simultaneously while testing those operationalisations and definitions available now. This might further this discussion and strengthen the related nursing practice. Our findings also indicate that further research aimed at strengthening nursing practice by developing, testing and implementing effective MoCs containing proactive preventative strategies directed at older people’s functional ability limitations and frailty is needed. This is important because we know the extent of negative health outcomes related to these conditions, which we can spare older people by preventing or reversing the states early [13].

### Evidence for practice

Regardless of the development of a research-based understanding, the main evidence for practice from our findings highlights the critical responsibilities of nurses in gaining a deeper understanding of older people’s experiences through their assessments and evaluations of functional ability limitations and frailty. Our findings imply that there is a distinct difference between older people’s and nurses’ perspectives of the state’s impact on everyday life and that of older individuals, with older people’s descriptions painting a more nuanced picture. To effectively implement a nursing practice that are capable of early detection and prevention of the often, subtle signs and symptoms of these states, it is essential for nurses to actively seek the perspectives of older people to provide care that is appropriately tailored to their needs. Nevertheless, our findings suggest that we still have a limited understanding, i.e., lacking knowledge, how nurses themselves view and experience functional ability limitations and frailty, particularly the former, as related or unrelated, or if they actively target them in their professional clinical practice in this context. Regardless, to be able to include the patient’s perspective into a tailored care demands systematic and evidence-based working modes. Example of the latter is models of care (MoCs) which outline, organise and guide nurses’ specific scope of practice, including their authorisation, responsibilities and functions. Thus, we suggest that MoCs need to emphasise the nurses’ clinical decision-making process**—**i.e., nursing process [98, 99]. Especially as it is central to all nursing practice and stresses the uniqueness of a nursing practice focusing on patients’ reactions to their health condition and their responses to treatment and nursing care rather than the patients actual medical diagnosis [cf. 100, 101]. Knowledge about the latter is essential since nurses’ clinical practice and decision-making processes are shaped by their competence and skills related to functional ability limitations and frailty. Thus, exploring nursing practice in relation to these states appears vital as more knowledge are warranted that can facilitate the development of feasible and acceptable MoCs for nursing practice in the ever-changing environments of home and facility-based care.

Currently, much practice in this context is characterised by task orientation and ‘firefighting’ rather than providing holistic care [102]. Our findings were related to a lack of formalised care plans, a lack of staffing, gaps in service, task-oriented services, time pressure, a lack of standardised assessment tools and a lack of training. These findings are also supported by previous research showing that nurses must more or less simultaneously address the factors related to the health care system, such as quality of care delivery and organisational and contextual factors [103, 104]. To address these challenges, it is crucial to develop nursing practice through both tangible clinical changes and research designed to yield appropriate, practical implications for nursing practice, in other words, research design to counteract waste of research and promote sustainable inquiry.

### Methodological considerations

There is an inherent strength in following Arksey and O’Malley’s [26] methodological framework. As such, it supported our iterative processes from the development of search strategies and the inclusion of full texts to the extraction and summarisation of the findings. The implementation of the PAGER framework [27] for the visualisation (patterning) and reporting of findings could also be viewed as a strength. Regardless of this—and together with our decision to aim for sensitivity instead of specificity [38] in our search strategies—we cannot exclude that some papers might have been missed. Despite recommendations [26] to not conduct critical appraisals in scoping reviews, we decided to assess both the quality and ethical considerations of the included papers. Lately, this has become a more common trait in scoping reviews, and by doing so, important perspectives can be revealed, particularly when the PAGER framework and its individual headings for reporting (i.e., ‘gaps and research recommendations’) are implemented. Assessing the ethical considerations made in the included papers may be a priority in scoping reviews (or, in that case, in all reviews). Others have implied that relevant information and/or gaps are likely to be identified [77, 105]. Our detailed description of our systematic process in stage 5—summarising—might be seen as a strength in relation to the analysis and reporting of our findings. Having opted to label the analysis as neither thematic nor content analysis, we leave it for the readers to evaluate its credibility [106].

## Implications and conclusions

The present scoping review, being the first strand in a tier of three further consecutive project strands [cf. 28], has enabled us to identify what is already well researched and where the possible gaps are, thus counteracting waste of research [107–109]. Together with these upcoming strands, these findings inform the development and testing of an intervention targeting nursing practice related to functional ability limitations and frailty in the context of long-term care. For example, we can conclude based on some of our findings that there is a need to develop interventions targeting nursing practice related to these conditions and that our main focus should be on RNs working in home-based care, highlighting and investigating both conditions as a central part of nurses’ scope of practice. There is also a need for the simultaneous research of functional ability limitations and frailty to achieve a more complete understanding as to develop knowledge supporting nursing practice and the nurse’s clinical competence in home- and facility-based care.

## Electronic supplementary material

Below is the link to the electronic supplementary material.

Supplementary Material 1: PRISMA-ScR checklist (pdf): Document showing filled out PRISMA-ScR checklist

Supplementary Material 2: PubMed search strategy Q1-Q3 (pdf): Document showing the search strategy for Q1-Q3 in PubMed

## Data Availability

The datasets underpinning the findings in this paper are available upon reasonable request.

## Abbreviations

RN: Registered nurse
MoC: Models of care
PRISMA-ScR: Preferred reporting items for systematic reviews and meta-analyses extension for scoping reviews
PICoS: Population, phenomenon of interest, context and study design
PAGER: Patterns, advances, gaps, evidence for practice and research recommendations
MeSH: Medical Subject Headings
CASP: Critical appraisal skills programme
ADLs: Activities of daily life
