# Supplementary material for: What do we know about nursing practice in relation to functional ability limitations, frailty and models of care among older people in home- and facility-based care: a scoping review
Source: BMC Nurs. 2025 Apr 10;24:406. doi: 10.1186/s12912-025-02948-7 (PMC11987274; doi:10.1186/s12912-025-02948-7)
Supplement: Supplementary file 2 — Supplementary Material 2: PubMed search strategy Q1-Q3 (pdf): Document showing the search strategy for Q1-Q3 in PubMed [file 12912_2025_2948_MOESM2_ESM.pdf]

Results from the search run 27.06.22

Search strategy for question 1: How is the condition of functional ability limitations among older people in home- or facility-based care described by key stakeholders?

**PubMed**	**Search block: Older people (P)**		**27.06.22**
**Search #**	**Query**	**Search string**	**Result**
#1	Aged	"aged"[MeSH Terms] OR "aged"[Title/Abstract]	3,846,902
#2	Elder*	"elder*"[Title/Abstract]	293,221
#3	Old/Older	"old"[Title/Abstract] OR "older"[Title/Abstract]	1,638,074
#4	Senior(s)	"Senior"[Title/Abstract] OR "Seniors"[Title/Abstract]	46,184
#5	Combination:	**#1 OR #2 OR #3 OR #4**	5,031,400

**PubMed**	**Search block: Significant others (P)**		**27.06.22**
**Search #**	**Query**	**Search string**	**Result**
#6	Significant other*	"significant other*"[Title/Abstract]	4,515
#7	Family/ Families	"Family"[MeSH Terms] OR "Family"[Title/Abstract] OR "Families"[Title/Abstract]	1,320,790
#8	Relative(s)	"Relative"[Title/Abstract] OR "Relatives"[Title/Abstract]	1,004,277
#9	Spouse(s)	"Spouses"[MeSH Terms] OR "Spouse"[Title/Abstract] OR "Spouses"[Title/Abstract]	26,445
#10	Partner(s)	"Partner"[Title/Abstract] OR "Partners"[Title/Abstract]	161,216
#11	Husband(s)	"Husband"[Title/Abstract] OR "Husbands"[Title/Abstract]	13,355
#12	Wife/ Wives	"Wife"[Title/Abstract] OR "Wives"[Title/Abstract]	11,350
#13	Next-of-kin	"next of kin*"[Title/Abstract]	1,690
#14	Combination:	**#6 OR #7 OR #8 OR #9 OR #10 OR #11 OR #12 OR #13**	2,387,952

PubMed	Search block: Nursing staff (P)		27.06.22
Search #	Query	Search string	Result
#15	Nurse(s)	"Nurses"[MeSH Terms] OR "Nurse"[Title/Abstract] OR "Nurses"[Title/Abstract]	335,842
#16	Nursing staff(s)	"nursing staff"[MeSH Terms] OR "nursing staff"[Title/Abstract] OR "nursing staffs"[Title/Abstract]	78,983
#17	Nursing personnel*	"nursing personnel*"[Title/Abstract]	2,814
#18	Nursing professional*	"nursing professional*"[Title/Abstract] OR "nurse professional*"[Title/Abstract]	3,073
#19	Nursing assistant*	"nursing assistants"[MeSH Terms] OR (("nursing"[Title/Abstract] OR "nurse"[Title/Abstract] OR "nurses"[Title/Abstract]) AND "assistant*"[Title/Abstract])	12,204
#20	Nursing aide(s)	("nursing"[Title/Abstract] OR "nurse"[Title/Abstract] OR "nurses"[Title/Abstract]) AND ("aide"[Title/Abstract] OR "aides"[Title/Abstract])	2,016
#21	Healthcare assistant*	"healthcare assistant*"[Title/Abstract] OR "health care assistant*"[Title/Abstract]	930
#22	Healthcare aide(s)	("healthcare"[Title/Abstract] OR "health care"[Title/Abstract]) AND ("aide"[Title/Abstract] OR "aides"[Title/Abstract])	896
#23	Home health aide(s)	"home health aides"[MeSH Terms] OR ("home health"[Title/Abstract] AND ("aide"[Title/Abstract] OR "aides"[Title/Abstract]))	1,068
#24	Care aide(s)	"Care"[Title/Abstract] AND ("aide"[Title/Abstract] OR "aides"[Title/Abstract])	1,915
#25	Care staff(s)	"Care staff"[Title/Abstract] OR "Care staffs"[Title/Abstract]	4,936
#26	Care assistant*	"Care assistant*"[Title/Abstract]	734
#27	Care personnel*	"Care personnel*"[Title/Abstract]	3,646
#28	Care provider*	"Care provider*"[Title/Abstract]	65,315
#29	Combination:	**#15 OR #16 OR #17 OR #18 OR #19 OR #20 OR #21 OR #22 OR #23 OR #24 OR #25 OR #26 OR #27 OR #28**	443,429

**PubMed**	**Search block: Population**		**27.06.22**
**Search #**	**Query**	**Search string**	**Result**
#30	Combination: Older people OR Significant others OR Nursing staff	**#5 OR #14 OR #29**	7,312,232

**PubMed**	**Search block: Functional ability limitations (Functional decline) (I)**		**27.06.22**
**Search #**	**Query**	**Search string**	**Result**
#31	Functional decline*	"functional decline*"[Title/Abstract] OR "declining function*"[Title/Abstract]	6,596
#32	Functional deterioration*	"functional deterioration*"[Title/Abstract] OR "deteriorating function*"[Title/Abstract] OR "deteriorated function*"[Title/Abstract]	1,758
#33	Functional impairment*	"functional impairment*"[Title/Abstract] OR "impaired function*"[Title/Abstract] OR "impairment in function*"[Title/Abstract] OR "functionally impaired"[Title/Abstract]	27,719
#34	Reduced functional independence/ Functional dependence	"Impaired functional independence"[Title/Abstract] OR "Reduced functional independence"[Title/Abstract] OR "Functional dependence"[Title/Abstract]	1,441
#35	Functional abilit*	"functional abilit*"[Title/Abstract]	7,768
#36	Functional disabilit*	"functional disabilit*"[Title/Abstract]	7,782
#37	Functional limitation*	"functional limitation*"[Title/Abstract]	7,847
#38	Functional capacit*	"reduced functional capacit*"[Title/Abstract] OR "impaired functional capacit*"[Title/Abstract]	516
#39	Functional loss	"Functional loss"[Title/Abstract]	2,901
#40	Functional status	"functional status"[MeSH Terms]	1,076
#41	Activities of daily li*/ daily living abilit*	"activities of daily li*"[Title/Abstract] OR "activity of daily li*"[Title/Abstract] OR "daily living activit*"[Title/Abstract] OR "daily living abilit*"[Title/Abstract]	36,731
#42	Combination:	**#31 OR #32 OR #33 OR #34 OR #35 OR #36 OR #37 OR #38 OR #39 OR #40 OR #41**	95,083

PubMed	Search block: Home- and facility-based care (Long-term care) (C)		27.06.22
Search #	Query	Search string	Result
#43	Long-term care	"long term care"[MeSH Terms] OR "long term care" [Title/Abstract]	41,673
#44	Community-dwelling	"community dwelling" [Title/Abstract]	28,677
#45	Community health nursing/ Community Nursing	"community health nursing" [MeSH Terms] OR "community health nursing" [Title/Abstract] OR "community nursing" [Title/Abstract]	21,509
#47	Elder care	"elder care"[Title/Abstract] OR "eldercare"[Title/Abstract]	1,127
#48	Housing for the elderly	"housing for the elderly"[MeSH Terms] OR "housing for the elderly"[Title/Abstract]	1,728
#49	Assisted living	"Assisted living" [Title/Abstract]	2,687
#50	Home care services	"home care services"[MeSH Terms] OR "home care"[Title/Abstract]	59,286
#51	Home health nursing	"home health nursing"[MeSH Terms] OR "home health nursing"[Title/Abstract]	548
#52	Nursing home	"nursing homes"[MeSH Terms] OR "nursing home*"[Title/Abstract]	55,454
#53	Care facility	"Care facility" [Title/Abstract] OR "Care facilities" [Title/Abstract]	26,224
#54	Residential (aged) care	"residential care" [Title/Abstract] OR "residential aged care" [Title/Abstract]	5,387
#55	Homes for the Aged	"homes for the aged"[MeSH Terms] OR "home for the aged"[Title/Abstract] OR "homes for the aged"[Title/Abstract]	14,901
#56	Combination:	**#43 OR #44 OR #45 OR #46 OR #47 OR #48 OR #49 OR #50 OR #51 OR #52 OR #53 OR #54 OR #55**	206,963

PubMed	Combination of search blocks		27.06.22
Search #	Query	Search string	Result
#57	Combination: (Population AND Context AND phenomenon of Interest) AND Limits	**(#30 AND #42 AND #56) AND Limits for: (2002/06/01:2022/06/30[Date - Publication] AND "english"[Language]) AND (Publication types)**	6,063

Search strategy for question 2: How is the condition of frailty among older people in home- or facility-based care described by key stakeholders?

**PubMed**	**Search block: Older people (P)**		**27.06.22**
**Search #**	**Query**	**Search string**	**Result**
#1	Aged	"aged"[MeSH Terms] OR "aged"[Title/Abstract]	3,846,902
#2	Elder*	"elder*"[Title/Abstract]	293,221
#3	Old/Older	"old"[Title/Abstract] OR "older"[Title/Abstract]	1,638,074
#4	Senior(s)	"Senior"[Title/Abstract] OR "Seniors"[Title/Abstract]	46,184
#5	Combination:	**#1 OR #2 OR #3 OR #4**	5,031,400

**PubMed**	**Search block: Significant others (P)**		**27.06.22**
**Search #**	**Query**	**Search string**	**Result**
#6	Significant other*	"significant other*"[Title/Abstract]	4,515
#7	Family/ Families	"Family"[MeSH Terms] OR "Family"[Title/Abstract] OR "Families"[Title/Abstract]	1,320,790
#8	Relative(s)	"Relative"[Title/Abstract] OR "Relatives"[Title/Abstract]	1,004,277
#9	Spouse(s)	"Spouses"[MeSH Terms] OR "Spouse"[Title/Abstract] OR "Spouses"[Title/Abstract]	26,445
#10	Partner(s)	"Partner"[Title/Abstract] OR "Partners"[Title/Abstract]	161,216
#11	Husband(s)	"Husband"[Title/Abstract] OR "Husbands"[Title/Abstract]	13,355
#12	Wife/ Wives	"Wife"[Title/Abstract] OR "Wives"[Title/Abstract]	11,350
#13	Next-of-kin	"next of kin*"[Title/Abstract]	1,690
#14	Combination:	**#6 OR #7 OR #8 OR #9 OR #10 OR #11 OR #12 OR #13**	2,387,952

PubMed	Search block: Nursing staff (P)		27.06.22
Search #	Query	Search string	Result
#15	Nurse(s)	"Nurses"[MeSH Terms] OR "Nurse"[Title/Abstract] OR "Nurses"[Title/Abstract]	335,842
#16	Nursing staff(s)	"nursing staff"[MeSH Terms] OR "nursing staff"[Title/Abstract] OR "nursing staffs"[Title/Abstract]	78,983
#17	Nursing personnel*	"nursing personnel*"[Title/Abstract]	2,814
#18	Nursing professional*	"nursing professional*"[Title/Abstract] OR "nurse professional*"[Title/Abstract]	3,073
#19	Nursing assistant*	"nursing assistants"[MeSH Terms] OR (("nursing"[Title/Abstract] OR "nurse"[Title/Abstract] OR "nurses"[Title/Abstract]) AND "assistant*"[Title/Abstract])	12,204
#20	Nursing aide(s)	("nursing"[Title/Abstract] OR "nurse"[Title/Abstract] OR "nurses"[Title/Abstract]) AND ("aide"[Title/Abstract] OR "aides"[Title/Abstract])	2,016
#21	Healthcare assistant*	"healthcare assistant*"[Title/Abstract] OR "health care assistant*"[Title/Abstract]	930
#22	Healthcare aide(s)	("healthcare"[Title/Abstract] OR "health care"[Title/Abstract]) AND ("aide"[Title/Abstract] OR "aides"[Title/Abstract])	896
#23	Home health aide(s)	"home health aides"[MeSH Terms] OR ("home health"[Title/Abstract] AND ("aide"[Title/Abstract] OR "aides"[Title/Abstract]))	1,068
#24	Care aide(s)	"Care"[Title/Abstract] AND ("aide"[Title/Abstract] OR "aides"[Title/Abstract])	1,915
#25	Care staff(s)	"Care staff"[Title/Abstract] OR "Care staffs"[Title/Abstract]	4,936
#26	Care assistant*	"Care assistant*"[Title/Abstract]	734
#27	Care personnel*	"Care personnel*"[Title/Abstract]	3,646
#28	Care provider*	"Care provider*"[Title/Abstract]	65,315
#29	Combination:	**#15 OR #16 OR #17 OR #18 OR #19 OR #20 OR #21 OR #22 OR #23 OR #24 OR #25 OR #26 OR #27 OR #28**	443,429

**PubMed**	**Search block: Population**		**27.06.22**
**Search #**	**Query**	**Search string**	**Result**
#30	Combination:<br>Older people OR<br>Significant others OR<br>Nursing staff	**#5 OR #14 OR #29**	7,312,232

**PubMed**	**Search block: Frailty (I)**		**27.06.22**
**Search #**	**Query**	**Search string**	**Result**
#31	Frailty	"frailty"[MeSH Terms] OR "frail elderly"[MeSH Terms] OR "frail*"[Title/Abstract]	35,498

**PubMed**	**Search block: Home- and facility-based care (Long-term care) (C)**		**27.06.22**
**Search #**	**Query**	**Search string**	**Result**
#32	Long-term care	"long term care"[MeSH Terms] OR "long term care" [Title/Abstract]	41,673
#33	Community-dwelling	"community dwelling" [Title/Abstract]	28,677
#34	Community health nursing/ Community Nursing	"community health nursing" [MeSH Terms] OR "community health nursing" [Title/Abstract] OR "community nursing" [Title/Abstract]	21,509
#35	Elder care	"elder care"[Title/Abstract] OR "eldercare"[Title/Abstract]	1,127
#36	Housing for the elderly	"housing for the elderly"[MeSH Terms] OR "housing for the elderly"[Title/Abstract]	1,728
#37	Assisted living	"Assisted living" [Title/Abstract]	2,687
#38	Home care services	"home care services"[MeSH Terms] OR "home care"[Title/Abstract]	59,286
#39	Home health nursing	"home health nursing"[MeSH Terms] OR "home health nursing"[Title/Abstract]	548
#40	Nursing home	"nursing homes"[MeSH Terms] OR "nursing home*"[Title/Abstract]	55,454
#41	Care facility	"Care facility" [Title/Abstract] OR "Care facilities" [Title/Abstract]	26,224
#42	Residential (aged) care	"residential care" [Title/Abstract] OR "residential aged care" [Title/Abstract]	5,387
#43	Homes for the Aged	"homes for the aged"[MeSH Terms] OR "home for the aged"[Title/Abstract] OR "homes for the aged"[Title/Abstract]	14,901

#44	Combination:	**#32 OR #33 OR #34 OR #35 OR #36 OR #37 OR #38 OR #39 OR #40 OR #41 OR #42 OR #43**	206,963

**PubMed**	**Combination of search blocks**		**27.06.22**
**Search #**	**Query**	**Search string**	**Result**
#45	Combination: (Population AND Context AND phenomenon of Interest) AND Limits	**(#30 AND #31 AND #44) AND Limits for: (2002/06/01:2022/06/30[Date - Publication] AND "english"[Language]) AND (Publication types)**	5,325

Search strategy for question 3: What models of care can be identified as targeting functional ability limitations or frailty in relation to older people in home- or facility-based care?

PubMed	Search block: Frailty (I)		27.06.22
**Search #**	**Query**	**Search string**	**Result**
#1	Frailty	"frailty"[MeSH Terms] OR "frail elderly"[MeSH Terms] OR "frail*"[Title/Abstract]	35,498

PubMed	Search block: Functional ability limitations (Functional decline) (I)		27.06.22
**Search #**	**Query**	**Search string**	**Result**
#2	Functional decline*	"functional decline*"[Title/Abstract] OR "declining function*"[Title/Abstract]	6,596
#3	Functional deterioration*	"functional deterioration*"[Title/Abstract] OR "deteriorating function*"[Title/Abstract] OR "deteriorated function*"[Title/Abstract]	1,758
#4	Functional impairment*	"functional impairment*"[Title/Abstract] OR "impaired function*"[Title/Abstract] OR "impairment in function*"[Title/Abstract] OR "functionally impaired"[Title/Abstract]	27,719
#5	Reduced functional independence/ Functional dependence	"Impaired functional independence"[Title/Abstract] OR "Reduced functional independence"[Title/Abstract] OR "Functional dependence"[Title/Abstract]	1,441
#6	Functional abilit*	"functional abilit*"[Title/Abstract]	7,768
#7	Functional disabilit*	"functional disabilit*"[Title/Abstract]	7,782
#8	Functional limitation*	"functional limitation*"[Title/Abstract]	7,847
#9	Functional capacit*	"reduced functional capacit*"[Title/Abstract] OR "impaired functional capacit*"[Title/Abstract]	516
#10	Functional loss	"Functional loss"[Title/Abstract]	2,901
#11	Functional status	"functional status"[MeSH Terms]	1,076
#12	Activities of daily li*/ daily living abilit*	"activities of daily li*"[Title/Abstract] OR "activity of daily li*"[Title/Abstract] OR "daily living activit*"[Title/Abstract] OR "daily living abilit*"[Title/Abstract]	36,731
#13	Combination:	**#2 OR #3 OR #4 OR #5 OR #6 OR #7 OR #8 OR #9 OR #10 OR #11 OR #12**	95,083

PubMed	Search block: Frailty (I) OR Functional ability limitations (Functional decline) (I)		27.06.22
**Search #**	**Query**	**Search string**	**Result**
#14	Combination: Frailty OR Functional ability limitations	**#1 OR #13**	130, 581

PubMed	Search block: Models of care (I)		27.06.22
**Search #**	**Query**	**Search string**	**Result**
#15	Nursing model	"models, nursing"[MeSH Terms] OR (("nursing"[Title/Abstract] OR "nurse"[Title/Abstract] OR "nurses"[Title/Abstract]) AND ("model"[Title/Abstract] OR "models"[Title/Abstract]))	52,573
#16	Care models	("Care"[Title/Abstract] OR "Caring"[Title/Abstract]) AND ("model"[Title/Abstract] OR "models"[Title/Abstract])	206,840
#17	Nursing frameworks	("nursing"[Title/Abstract] OR "nurse"[Title/Abstract] OR "nurses"[Title/Abstract]) AND ("framework"[Title/Abstract] OR "frameworks"[Title/Abstract])	16,314
#18	Nursing guidelines	"nursing guideline*"[Title/Abstract] OR "nursing care guideline*"[Title/Abstract]	209
#19	Care frameworks	("Care"[Title/Abstract] OR "Caring"[Title/Abstract]) AND ("framework"[Title/Abstract] OR "frameworks"[Title/Abstract])	51,359
#20	Nursing interventions	("nursing"[Title/Abstract] OR "nurse"[Title/Abstract] OR "nurses"[Title/Abstract]) AND ("intervention"[Title/Abstract] OR "interventions"[Title/Abstract])	60,844
#21	Care interventions	("Care"[Title/Abstract] OR "Caring"[Title/Abstract]) AND ("intervention"[Title/Abstract] OR "interventions"[Title/Abstract])	254,767
#22	Nurse-led	"nurse led"[Title/Abstract] OR "nursing led"[Title/Abstract] OR "led by nurs*"[Title/Abstract]	4,775
#23	Patient pathway	("patient*"[Title/Abstract] AND "pathway*"[Title/Abstract])	225,970
#24	Care pathway	"care pathway*"[Title/Abstract]	6,350
#25	Critical pathway	"critical pathways"[MeSH Terms] OR "critical pathway*"[Title/Abstract]	9,210
#26	Clinical pathway	"clinical pathway*"[Title/Abstract]	4,296
#27	Care map	"care map*"[Title/Abstract]	304
#28	Critical path	"critical path"[Title/Abstract] OR "critical paths"[Title/Abstract]	620

#29	Clinical path	"clinical path"[Title/Abstract] OR "clinical paths"[Title/Abstract]	260
#30	Care path	"care path"[Title/Abstract] OR "care paths"[Title/Abstract]	343
#31	Care guideline	"guidelines as topic"[MeSH Terms] OR "guideline"[Publication Type] OR "care guideline*"[Title/Abstract]	209,676
#32	Practice guideline	"practice guidelines as topic"[MeSH Terms] OR (("practice"[Title/Abstract] OR "practical"[Title/Abstract]) AND "guideline*"[Title/Abstract])	205,596
#33	Evidence based guideline	"evidence based guideline*"[Title/Abstract]	9,801
#34	Clinical guideline	"clinical guideline*"[Title/Abstract]	16,649
#35	Clinical protocol	"clinical protocols"[MeSH Terms] OR "clinical protocol*"[Title/Abstract]	189,595
#36	Care protocol	"care protocol*"[Title/Abstract]	2,059
#37	Nursing protocol	"nursing protocol*"[Title/Abstract]	200
#38	Evidence based recommendation*	"evidence based recommendation*"[Title/Abstract]	5,782
#39	Combination:	**#15 OR #16 OR #17 OR #18 OR #19 OR #20 OR #21 OR #22 OR #23 OR #24 OR #25 OR #26 OR #27 OR #28 OR #29 OR #30 OR #31 OR #32 OR #33 OR #34 OR #35 OR #36 OR #37 OR #38**	1,167,121

**PubMed**	**Search block: Home- and facility-based care (Long-term care) (C)**		**27.06.22**
**Search #**	**Query**	**Search string**	**Result**
#40	Long-term care	"long term care"[MeSH Terms] OR "long term care" [Title/Abstract]	41,673
#41	Community-dwelling	"community dwelling" [Title/Abstract]	28,677
#42	Community health nursing/ Community Nursing	"community health nursing" [MeSH Terms] OR "community health nursing" [Title/Abstract] OR "community nursing" [Title/Abstract]	21,509
#43	Elder care	"elder care"[Title/Abstract] OR "eldercare"[Title/Abstract]	1,127
#44	Housing for the elderly	"housing for the elderly"[MeSH Terms] OR "housing for the elderly"[Title/Abstract]	1,728
#45	Assisted living	"Assisted living" [Title/Abstract]	2,687
#46	Home care services	"home care services"[MeSH Terms] OR "home care"[Title/Abstract]	59,286
#47	Home health nursing	"home health nursing"[MeSH Terms] OR "home health nursing"[Title/Abstract]	548

#48	Nursing home	"nursing homes"[MeSH Terms] OR "nursing home*"[Title/Abstract]	55,454
#49	Care facility	"Care facility" [Title/Abstract] OR "Care facilities" [Title/Abstract]	26,224
#50	Residential (aged) care	"residential care" [Title/Abstract] OR "residential aged care" [Title/Abstract]	5,387
#51	Homes for the Aged	"homes for the aged"[MeSH Terms] OR "home for the aged"[Title/Abstract] OR "homes for the aged"[Title/Abstract]	14,901
#52	Combination:	**#40 OR #41 OR #42 OR #43 OR #44 OR #45 OR #46 OR #47 OR #48 OR #49 OR #50 OR #51**	206,963

**PubMed**	**Combination of search blocks**		**27.06.22**
**Search #**	**Query**	**Search string**	**Result**
#53	Combination: (Population AND Context AND phenomenon of Interest) AND Limits	**(#14 AND #39 AND #52) AND Limits for: (2002/06/01:2022/06/30[Date - Publication] AND "english"[Language] AND "aged"[MeSH Terms]) AND (Publication types)**	2,785
